# Occurrence of mycotoxins in fish feed and its consequences for aquaculture with special regard to the role of insect products

**DOI:** 10.1007/s12550-025-00628-8

**Published:** 2026-01-22

**Authors:** Mikołaj Bittner, Paweł Brzuzan, Maciej Woźny

**Affiliations:** Department of Environmental Biotechnology, Institute of Environmental Engineering, Faculty of Geoengineering, University of Warmia and Mazury in Olsztyn, ul. Słoneczna 45G, 10-709 Olsztyn, Poland

**Keywords:** Alternative protein, Emerging mycotoxins, Feed safety, Fish health and welfare, Sustainable aquaculture

## Abstract

Infection of plants with molds and inadequate storage conditions of plant materials contribute to contamination of crops like cereals, corn and soybeans with mycotoxins. Plant-derived ingredients serve as major protein sources in aquaculture feeds; however, the increasing use of insects as alternative nutrient sources introduces additional potential pathways for mycotoxin contamination. Strong evidence shows that exposure of fish to mycotoxins can negatively affect their health, leading to reduced growth, impaired immune response and reproductive disorders. However, knowledge of the effects of mycotoxins on farmed insects is still limited. Little is known about the insect metabolism of mycotoxins and their transfer to animal feed, which constitutes a weakness of safety assessments of insects as feed components. This review provides comprehensive information about the consequences of mycotoxin contamination of feed materials and feeds used in aquaculture. To facilitate understanding of the problem and its scale, the mycotoxin levels in plant materials and finished feeds used in aquaculture were compiled and analyzed together with the contamination levels of fish-derived food. Moreover, the available data on the effects of fish exposure to the selected mycotoxins are summarized, highlighting the complexity of the biological activity of these compounds. Finally, recent challenges to the safety of using insects as a protein source in fish nutrition are discussed. The information provided herein is important for assessing the health and economic risks of the growing use of modern feed ingredients in the sustainable aquaculture.

## Introduction

Mycotoxins are harmful metabolites produced by mold fungi that are commonly found in the environment. Infection of crops by molds in field and inadequate storage conditions contribute to widespread mycotoxin contamination of agricultural crops such as cereals, corn and soybeans (DSM-Firmenich, 2024). Plant feed materials have long been used in aquaculture as a source of protein for feeding various fish used in aquaculture, including both omnivorous and carnivorous fish species. However, the presence of mold on plant material used to produce fish feed can be considered as a potential source of mycotoxins, posing threats to aquaculture safety (Aragão et al. 2022). Growing body of evidence now demonstrates that mycotoxins can negatively affect fish health, leading to reduced growth, impaired immune response and reproductive disorders (Cimbalo et al. 2020; Oliveira And Vasconcelos 2020). Importantly, however, plant feed components used as a source of nutrients are not the only possible source of mycotoxin contamination in fish feed.

Recently, in the European Union, selected insect species (like black soldier fly or yellow mealworm larvae) can be used as raw material for the production of feed for selected animals, including fish reared in aquaculture (EU Commission Regulation 2017/893 of May 24 2017). However, the production of insects for feed materials – oil and protein involves some restrictions on their feeding, i.e., the insects cannot be fed with certain products obtained from other animals, like meat or bone meal of other farmed animals. As a consequence, insects are thought to be used to convert plant materials with low digestibility into high-quality, easily-digestible protein (Alfiko et al. 2022). Moreover, using low-quality feedstuffs or by-products from agriculture and the food industry to feed the insects may be considered as an attractive solution to reduce food waste (Maroušek et al. 2023). However, such materials can be molded and contain significant amounts of mycotoxins, potentially increasing risks to aquaculture safety.

**Fig. 1:**
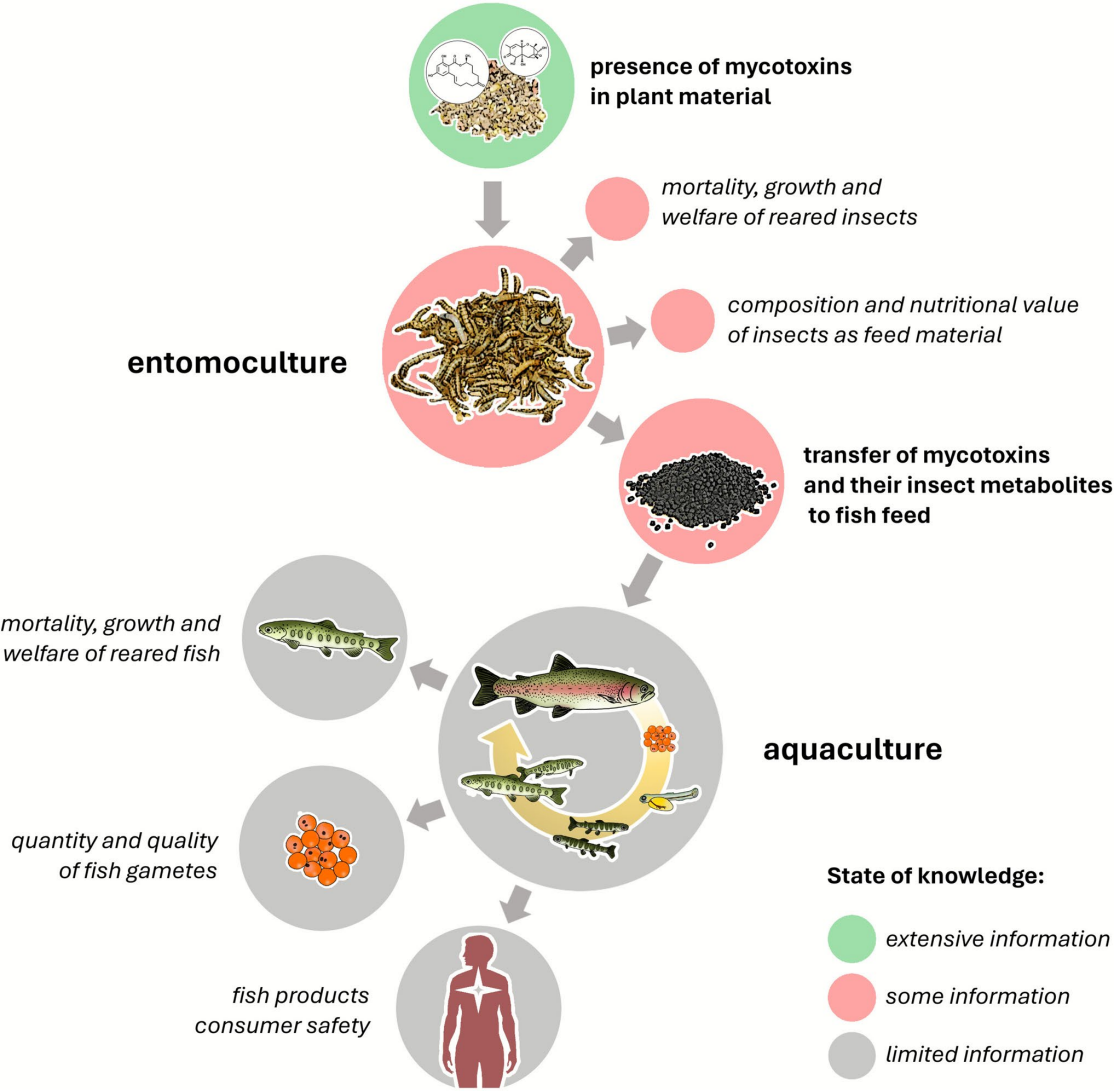
Potential consequences for aquaculture associated with the presence of mycotoxins in plant material and diet of insects used as a protein source in fish feed

Despite advancing insect-farming industry, knowledge of the effects of mycotoxins on farmed insects is still limited. It is known that the presence of mycotoxins in the diet of insect larvae does not markedly affect their growth or mortality, however, there is a lack of detailed information on the metabolism of many mycotoxins by insects and the possibility of their transfer to fish feed (Bisconsin-Junior et al., 2023; Niermans et al. 2024). If some mycotoxins had the ability to accumulate in insect bodies, their presence in the diet of larvae could potentially affect the growth, health and reproduction of fish fed feed containing the insect protein or oil. Moreover, in the case of cross-contamination, mycotoxins (or their metabolites produced by insects) could pose a risk to fish consumers (Fig. 1). Currently, the scientific literature does not include any studies involving fish fed feed containing insects reared on substrates contaminated with any mycotoxins. The lack of knowledge in this regard is a noticeable gap in the assessment of the safety of using insects in animal feed.

The aim of this review is to provide a comprehensive information about the risks associated with the presence of mycotoxins in plant materials and feed used in aquaculture. To facilitate understanding of the scale of the problem, the first section presents the definition and classification of mycotoxins and addresses the levels of contamination with these compounds of various plant raw materials and finished feeds used in fish farming, as well as fish-derived products. The next section compiles the available data on the effects of fish exposure to selected mycotoxins, demonstrating the complexity of the biological activity of these compounds and possible consequences of their presence in feed. The final part of the paper is focused on recent challenges to the safety of using insects as a protein source in fish nutrition. The information summarized and discussed in this review is crucial for assessing the health and economic consequences of the growing use of plant materials and insects as feed ingredients in modern, sustainable aquaculture. This review was prepared based on an extensive literature search conducted with scientific databases such as Scopus, using combinations of keywords related to “mycotoxins”, “aquaculture”, “fish feed”, “insects”, and common synonyms.

## Mycotoxin classification

Mycotoxins are secondary metabolites of microscopic filamentous fungi commonly known as molds (Bennett and Klich 2003). Molds that produce mycotoxins are widespread, thriving on diverse substrates across various environmental conditions. In agricultural commodities, the extent of crop contamination with these toxins typically fluctuates annually, as it is influenced by the weather and other environmental variables (Bennett and Klich 2003; DSM-Firmenich, 2024).

Over the past ten years, as scientists have issued more frequent warnings about global warming, they have connected it to an increase in the prevalence of mycotoxin-producing molds in diverse geographical regions worldwide (Kos et al. 2023). As climate change becomes more pronounced in the future, it may modify host resilience and host–pathogen interactions, significantly influencing the development of toxigenic molds. The present climate scenario urges the need for not only better diagnostic tools but also a deeper understanding of the biological characteristics of agricultural cultivars and toxigenic molds (Casu et al. 2024).

The classification of mycotoxins presents a challenge due to their diverse chemical structures, varied biosynthetic origins, numerous biological effects, and production by a broad array of fungal species (Janik et al. 2021) Scientists often evaluate the threats that these toxins pose based on their occurrence, risk factors influencing exposure of humans and animals, and standards of practice for limiting exposure. Scientists often evaluate the risks associated with mycotoxins through structured risk assessment, which includes hazard identification, hazard characterization, exposure assessment, and risk characterization.

This has led to the use of the terms “mycotoxins of most concern” and “emerging mycotoxins”, which are explained in the following sections. Additionally, the term “masked mycotoxins” is treated in Sect. "Masked mycotoxins".

### Mycotoxins of most concern

Several mycotoxins are of significant concern, mostly due to their great potential to pose serious health risks, but also due to their widespread occurrence in various crops and plant products. Many sensitive analytical methods are available to detect these compounds, and the literature describing their harmful effects on animals is very extensive. These toxins include some of the most well-known and well-studied mycotoxins like aflatoxins (AFs), fumonisins (FUMs), fusariotoxins, and ochratoxins.

AFs are produced by certain members of the genus *Aspergillus*, particularly *A. flavus* and *A. parasiticus*, which are known to contaminate crops such as peanuts, cereals, and tree nuts. These substances are genotoxic carcinogens, inducing liver tumors in both animals and humans (Pickova et al. 2021). Among them, aflatoxin B1 (AFB_1_) is the most potent carcinogen, and the majority of the available toxicological data pertain to this particular substance (Rushing and Selim 2019).

*Fusarium* molds produce a number of chemically-diverse mycotoxins, collectively termed fusariotoxins. FUMs like fumonisin B1 (FB_1_) or fumonisin B2 (FB_2_) are considered among the most important; they are produced by *Fusarium verticillioides*, which is the most commonly reported fungal species that infects maize (*Zea mays*). These mycotoxins can cause various health issues, including neural tube defects in animals (Kamle et al. 2019). *Aspergillus niger* can also produce FUMs and is a common contaminant of food worldwide, causing a disease known as “black mold” on certain fruits and vegetables, such as grapes, apricots, onions, and peanuts (Alegbeleye et al. 2022).

Another fusariotoxin (and a trichothecene group member) is deoxynivalenol (DON), also known as vomitoxin, which is associated primarily with *Fusarium graminearum*, a species that commonly contaminates grains like wheat, barley, and corn. This mycotoxin can cause vomiting, reduce food intake, and suppress the immune response in livestock (Sumarah 2022).

A third fungal metabolite classified as the mycotoxin of concern is zearalenone (ZEN), which was initially detected in *Fusarium graminearum*. Further studies revealed that it is also produced by other *Fusarium* genera, including* F. culmorum, F. cerealis*, *F. equiseti*,* F. verticillioides*, and* F. incarnatum* and that it contaminates grains such as corn, wheat, rice, and oats (Liu and Applegate 2020). In animals, the most pronounced effect of ZEN and its metabolites is dysfunction of the reproductive system due to its estrogenic properties (Ropejko and Twarużek, 2021).

Ochratoxins are produced by *Aspergillus* (mainly *A. ochraceus* and *A. carbonarius*, also 33% of industrial strains of *A. niger*) and *Penicillium* (especially *P. verrucosum*) (Pfohl-Leszkowicz and Manderville 2007). Ochratoxin A (OTA) in particular, which among OTAs has drawn the most attention, has been found to contaminate cereals, coffee, and wine. It has been linked to kidney damage and is a potential carcinogen in mice and rats (Tao et al. 2018). As this mycotoxin and the AFs pose the greatest risk to public health, their occurrence is closely regulated by the European Union, i.e. by the Commision’s Directive 2002/32/EC or Regulation (EU) 2023/915. The occurrence of other mycotoxins of concern (i.e., FUMs, DON, ZEN) is addressed through recommended guideline values that help to minimize contamination risks, such as those in the Commission Recommendation 2006/576/EC.

### Emerging mycotoxins

“Emerging mycotoxins” refers to mycotoxins that have been newly identified or are receiving increased attention due to advancements in analytical techniques, improved detection methods, and expanded research efforts. The designation “emerging” does not mean that these mycotoxins are newly produced by fungi; instead, it reflects an increased awareness of their presence and potential risks. These mycotoxins may not have been well-studied or regulated in the past, and their significance in terms of occurrence, toxicity, and effects on human and animal health is becoming better understood.

Some examples of emerging mycotoxins are the enniatins (ENNs) and beauvericin (BEA); these are cyclic peptides that display ionophoric activity, which is responsible for their multifaceted biological effects. These substances are produced by *Fusarium* fungi, although BEA can be also produced by *Beauveria bassiana* (Yin et al. 2022)*.* Another example of an emerging mycotoxin is moniliformin (MON), which is formed in cereals and grains by several *Fusarium* species including *F. moniliforme, F. avenaceum, F. subglutinans, F. proliferatum,* and* F. fujikuroi* (Agriopoulou et al. 2020). MON has a simpler chemical structure than ENNs and BEA, and it mainly acts as an enzymatic inhibitor of the Krebs cycle, impairing cellular energy supply (Fremy et al. 2019).

Recently, toxins produced by *Alternaria* molds have also been categorized as emerging mycotoxins. These mycotoxins are a chemically diverse group of compounds, and they include genotoxins like alternariol (AOH) and its monomethyl ether (AME), which are commonly produced by *Alternaria alternata* (Aichinger et al., 2021a). Despite decades of academic research on the occurrence and toxicity of the *Alternaria* toxins, no regulations have been enacted. However, ongoing research in this field has been dynamic, prompting the European Food Safety Authority to consistently request additional data (Aichinger et al., 2021b; Fraeyman et al. 2017; Lattanzio et al. 2022).

Climate change, including changes in factors such as temperature, available water, and light quality/quantity, and more commonly experienced extreme conditions like drought, desertification, and variations in humid/dry cycles, stands out as the most crucial factor influencing the stages of the fungal life cycle. It affects their capacity to colonize crops, survive, and produce toxins. The response of mycotoxigenic fungi to climate change has the potential to cause a shift in their geographical distribution and affect the patterns in which mycotoxins occur (Zingales et al. 2022). The advancement of research, modern detection methods, and the increasing influx of data from various parts of the world may soon lead to the reclassification of certain “emerging mycotoxins” as "mycotoxins of most concern" (Aichinger et al., 2021b). Consequently, some of these mycotoxins may become the subject to regulations within the European Union.

### Masked mycotoxins

The term “masked mycotoxins” was first coined by Gareis et al., who demonstrated that ZEN-14-glucoside (ZEN-14Glc) could undergo hydrolysis, releasing free ZEN during swine digestion (Gareis et al. 1990). Initially, masked mycotoxins were considered to be toxins not detectable through traditional analytical techniques. However, to prevent confusion, it is now recommended to use the term “masked mycotoxins” exclusively for plant-conjugated biologically modified phase II metabolites, as routine analyses have difficulties detecting all matrix-associated, biologically, and chemically altered mycotoxins (Berthiller et al. 2013). Masked mycotoxins that conform to this definition are produced via plant defense mechanisms involving glucosylation catalyzed by uridine diphosphate-glucosyltransferases (Zhang et al. 2020).

Documentation of the presence of masked mycotoxins, specifically mycotoxin-glucosides, initially centered around deoxynivalenol 3-glucoside (DON-3Glc) and zearalenone 4-glucoside (ZON-4Glc) (Berthiller et al. 2005; Schneweis et al. 2002). Currently, however, glucose conjugates like DON-3Glc, T-2-toxin-3-glucoside (T-2-3Glc), HT-2-toxin-3-glucoside (HT-2-3Glc), nivalenol-3-glucoside (NIV-3Glc), ZEN-14Glc, α-zearalenol-14-glucoside (α-ZEL-14Glc), and β-zearalenol-14-glucoside (β-ZEL-14Glc) are among the most commonly reported modified mycotoxins found in various plant materials (Freire and Sant’Ana 2018).

Masked mycotoxins exhibit significantly lower toxicity than their free parent compounds. This is mostly due to the fact that the chemical structures of these substances tend to be stable in the upper gastrointestinal tract, and their intestinal absorption is markedly lower than that of free mycotoxins (Gratz 2017). However, masked mycotoxins can be successfully converted back into their parent compounds by microorganisms inhabiting the other sections of the digestive system, like the intestines or the rumen. For example, DON-3Glc is relatively inert, usually resisting small-intestinal digestion, and does not get absorbed by epithelial cells into systemic circulation. However, in some cases, it can be easily converted into its free parent compound, being efficiently hydrolyzed into free DON by gut microbiota (Berthiller et al. 2011; De Nijs et al. 2012). On the other hand, ZEN-14Glc appears more accessible to the gut microbiota, commonly undergoing hydrolysis to free ZEN and further metabolism to known metabolites (e.g. via hydroxylation to α- and β-zearalenol) and to as-yet-undetermined metabolites (Gratz et al. 2017).

Elevated proportions of masked mycotoxins coexisting with their parent forms in diverse cereal-based food and animal feed have the potential to significantly amplify overall exposures, introducing additional health risks for both humans and animals (Zhang et al. 2020). However, our knowledge concerning the presence of these compounds, the means by which they are metabolized in animals, and their biological effects is still fragmentary.

Increasing our understanding of masked mycotoxins has the potential to influence on our perception of the threat they pose, leading to changes in regulatory framework of the European Union. It may transpire that plant-modified mycotoxins, after hydrolysis in the gastrointestinal system of humans and animals, elevate the levels of well-known mycotoxins of greatest concern. This could expose animals and consumers to the harmful effects of these compounds. Thus, a better understanding of this phenomenon could be crucial for public health.

## Occurrence of mycotoxins and their main sources in aquaculture

In the traditional view of aquaculture, the main source of protein in fish feed is fishmeal obtained from marine catches of fish, mainly from species with a high proportion of oil and bones. However, the rapid development of aquaculture coupled with the decline in catches from natural stocks has led to a decrease in the availability of these marine ingredients and an increase in their prices. These difficult market conditions have prompted feed manufacturers to look for alternative protein sources such as soybean meal (Glencross et al. 2024). Furthermore, as discussed later in this review (see Chapter 5), it has recently become evident that plant-based feed materials are not the only viable substitutes for conventional marine proteins. Other promising alternatives include insect meals; single-cell proteins derived from fungi, yeasts and bacteria; as well as microalgae (Fernandes et al. 2024).

As the presence of molds in plant material used as a protein source in fish feed can be a source of mycotoxins, this situation can be considered a potential threat to aquaculture safety. Therefore, the following subsections of this review compile and analyze the available results on mycotoxin contamination of plant materials and finished fish feed to create a picture of the possible consequences for fish consumers. Readers who wish to compare the levels of mycotoxins detected in feeds (Sect. "Presence of mycotoxins in fish feed") with effective doses reported in in vivo studies (Sect. "Effects of mycotoxins on aquaculture fish species") can make use of the relevant tables, with the caveat that it should be remembered that direct quantitative comparisons remain challenging, due to differences in fish species, feed composition, and co-occurrence of mycotoxins between the cited studies.

### Presence of mycotoxins in raw materials

The presence of mycotoxins in raw materials used for fish feeding or fish feed production is a widespread concern due to their detrimental effects on animal health. The literature indicates significant variation in mycotoxin contamination levels depending on the region and the raw material (Table 1). This review examines these differences, highlights regions with the highest and lowest contamination, and explores potential causes for the trends. It should be noted that most of the cited studies report mycotoxin levels in raw plant commodities rather than in ingredients actually used in aquaculture feeds. Consequently, the reported levels may not directly reflect concentrations in processed feed components, such as soy protein concentrate or wheat gluten.

**Table 1:** Occurrence of selected mycotoxins in raw materials used to produce fish feed

Mycotoxin	Material (EU limit/recommendation*)	Country/region	Content range (µg/kg)	Average content (µg/kg)	Reference
AFB_1_	DGGS (20 μg/kg)	China	≥ 0.5–13.6	10.4	Wu et al. 2016
	Maize (20 μg/kg)	Kenya	1.0–1137.4	16.0	Sirma et al. 2016
		China	0.5–300.0	47.9	Liu and Applegate 2020
			0.5–25.5	3.9	Abdou et al. 2017
		Norway	0.13–100.4	31.1	Bernhoft et al. 2016
		Serbia	1.3–88.8	11.4	Hajnal et al. 2017
		Iran	0–45.46	9.94	Hashemi 2016
		Brazil	0.49–6.5	NM	Savi et al. 2016
	Soybean (20 μg/kg)	Brazil	LOQ – 7.9	0.5	Calori-Domingues et al. 2018
	Soybean meal (20 μg/kg)	Pakistan	0.09–105.9	4.9	Iqbal et al. 2016
		Iran	NM – 11.46	6.62	Hashemi 2016
		China	≥ 0.5–9.8	3.9	Wu et al. 2016
	Wheat (20 μg/kg)	China	0.55–4.5	11	Liu et al. 2016
		Pakistan	0.05–4.78	0.51	Asghar et al. 2016
	Wheat bran (20 μg/kg)	Iran	0–56.13	2.94	Hashemi 2016
		China	0.5–10.9	2.6	Wu et al. 2016
DON	Maize (8000 μg/kg)	South Korea	≥ 3.3–232.56	190.78	Kim et al. 2016
		China	≥ 100–4320.9	755.1	Wu et al. 2016
		China	≥ 100–19,811.0	13,394.4	Liu et al. 2016
		Serbia	252.3–6280	921.1	Kos et al. 2017
		Poland	≥ 1.0–6688.0	766	Kosicki et al. 2016
	DDGS (NM)	China	≥ 100–2146.8	1319.5	Wu et al. 2016
	Wheat (8000 μg/kg)	Norway	5.0–94.0	28.3	Bernhoft et al. 2016
		China	≥ 100–3536.2	12.62	Liu et al. 2016
		Belgium/Hungary	0–1113.0	244	Sanders et al. 2016
	Wheat bran (8000 μg/kg)	China	≥ 100–3503.2	951.2	Wu et al. 2016
	Wheat grains (8000 μg/kg)	Slovakia	NM – 5100.0	740.0	Šliková et al. 2016
	Winter wheat (8000 μg/kg)	Lithuania	≥ 100–1393.0	383.98	Supronienė et al. 2016
	Soybean meal (NM)	China	≥ 100–786.4	457.5	Wu et al. 2016
FB_1_	Corn grain (60,000 µg/kg)	Brazil	16–1732.0	289.0	Savi et al. 2016
	Corn grits (60,000 µg/kg)	Brazil	88–2727	719.0	Savi et al. 2016
	Corn meal (60,000 µg/kg)	Brazil	75–5439.0	1305.0	Savi et al. 2016
	Maize (60,000 µg/kg)	Tanzania	63.26–213.15	157.88	Magembe et al. 2016
		Norway	31–8750.0	1001	Bernhoft et al. 2016
	Ground maize (60,000 µg/kg)	South Africa	26.036–379.242	147.236	Changwa et al. 2018
	Maize kernel (60,000 µg/kg)	China	≥ 4–28,285	1878	Guo et al. 2016
	Maize (60,000 µg/kg)	Egypt	1–2453	NM	Abdallah et al. 2017
	Soybean powder (NM)	Nigeria	NM – 4286.0	NM	Egbuta et al. 2016
	Soybean seeds (NM)	Nigeria	33–2270	NM	Egbuta et al. 2016
FB_2_	Corn grain (60,000 µg/kg)	Brazil	32–743	254	Savi et al. 2016
	Corn grits (60,000 µg/kg)	Brazil	48–1454	386	Savi et al. 2016
	Corn meal (60,000 µg/kg)	Brazil	52–1481	651	Savi et al. 2016
	Ground maize (60,000 µg/kg)	South Africa	26.036–379.242	147.236	Changwa et al. 2018
	Maize Kernel (60,000 µg/kg)	China	≥ 3–11,809.0	853	Guo et al. 2016
	Maize (60,000 µg/kg)	Norway	5–3540	354	Bernhoft et al. 2016
OTA	Maize (250 µg/kg)	Poland	≥ 0.13–86.0	NM	Kosicki et al. 2016
		Qatar	NM – 350.0	181.0	Hassan et al. 2018
	Wheat/wheat bran (250 µg/kg)	Qatar	NM – 45	3	Hassan et al. 2018
	Maize (250 µg/kg)	South Africa	> LOD – 95	NM	Gruber-Dorninger et al. 2018
		Egypt	2.8–11	NM	Abdallah et al. 2017
	Soybean powder (NM)	Nigeria	NM – 125	NM	Egbuta et al. 2016
	Soy bean meal (NM)	Pakistan	4.33–211.16	113.43	Abidin et al. 2017
	Soybean seeds (NM)	Nigeria	NM – 51	NM	Egbuta et al. 2016
ZEN	Maize (2000 µg/kg)	China	≥ 10–1442.5	251.5	Wu et al. 2016
		China	≥ 10–1613.7	260.6	Liu et al. 2016
		Serbia	260.4–9050.0	363.3	Kos et al. 2017
		Egypt	0.46–184.0	NM	Abdallah et al. 2017
		Poland	≥ 0.07–521.0	75.3	Kosicki et al. 2016
	Wheat (2000 µg/kg)	China	≥ 10–1278.9	215.0	Liu et al. 2016
	Wheat bran (2000 µg/kg)	China	≥ 10–329.0	148.1	Wu et al. 2016
	Soybean (NM)	Brazil	LOQ – 104.0	16.7	Calori-Domingues et.al 2018
		Pakistan	0.15–120.89	18.90	Iqbal et al. 2016
		China	≥ 10–332.5	189.5	Wu et al. 2016

^*^EU limits/recommendations according to DIRECTIVE 2002/32/EC OF THE EUROPEAN PARLIAMENT AND OF THE COUNCIL of 7 May 2002 on undesirable substances in animal feed (EC, 2002); COMMISSION RECOMMENDATION of 17 August 2006 on the presence of deoxynivalenol, zearalenone, ochratoxin A, T-2 and HT-2 and fumonisins in products intended for animal feeding (EC, 2006)

The table clearly shows that levels of mycotoxin contamination in raw materials differ considerably between regions (Sirma et al. 2016; Liu et al. 2016; Bernhoft et al., 2017; Janić Hajnal et al. 2017). For example, considering AFB_1_ contamination, one of the most toxic mycotoxins, maize samples from Kenya exhibited a wide range of contamination levels, from 1.0 to 1137.4 µg/kg, with an average value of 16.0 µg/kg (Sirma et al. 2016), which is near the European Union’s threshold of 20 µg/kg for AFs in animal feed. In other regions the contamination level is even higher; for example, China where contamination in maize ranged from 0.5 to 300.0 µg/kg, with an average of 47.9 µg/kg (J. Liu et al. 2016). Similarly, contamination in Norway ranged from 0.13 to 100.4 µg/kg, with an average of 31.1 µg/kg (Bernhoft et al. 2016), indicating a high average level of contamination compared to the extreme values in Kenya. Finally, Brazil consistently showed the lowest contamination levels across various materials, with soybeans being a notable example, where contamination ranged from below the limit of quantification (LOQ) to a mere 7.9 µg/kg (Calori-Domingues et al. 2018).

For DON, another prevalent mycotoxin, the disparity between regions is even more striking. In China, DON levels in maize varied widely, with a maximum of 19,811.0 µg/kg and an average of 13,394.4 µg/kg (J. Liu et al. 2016), far exceeding the European Union recommendation of 8000 µg/kg for this toxin. Other regions, like Serbia, displayed moderately high contamination levels, with an average of 921.1 µg/kg and a range from 252.3 to 6280 µg/kg (Kos et al. 2023). Similarly, Poland reported a range of contamination levels, from 1.0 to 6688.0 µg/kg, and an average of 766 µg/kg (Kosicki et al. 2016). In contrast, Norway, with DON levels ranging from 5.0 to 94.0 µg/kg and an average of only 28.3 µg/kg in wheat, had much lower contamination, illustrating marked regional differences (Bernhoft et al. 2016).

The contamination of raw materials with ZEN displayed similar variation. In maize, Serbia had the highest reported contamination, with values ranging from 260.4 to 9050.0 µg/kg and an average of 363.3 µg/kg (Kos et al. 2017), well above levels seen in Poland (0.07 to 521.0 µg/kg, averaging 75.3 µg/kg) (Kosicki et al. 2016) and Egypt (0.46 to 184.0 µg/kg) (Abdallah et al. 2017). China, another key region for agricultural production, showed moderately high levels, with an average of 251.5 µg/kg in maize and 215.0 µg/kg in wheat (J. Liu et al. 2016; L. Wu et al. 2016), which, while significant, are still lower than the values in Serbia.

Fumonisins (FB_1_ and FB_2_) exhibited similar regional disparities. In Brazil, corn meal had FUMs levels ranging from 75 to 5439.0 µg/kg, with an average of 1305.0 µg/kg (Savi et al. 2016), which was relatively moderate. However, in China, maize kernels had much higher contamination, ranging from 4 to 28,285 µg/kg, with an average of 1878 µg/kg (Guo et al. 2016). On the other hand, Norway reported much lower contamination levels, with values ranging from 31 to 8750 µg/kg and an average of 1001 µg/kg (Bernhoft et al. 2016).

Lastly, OTA contamination, although less widely distributed, displayed significant regional differences as well. Qatar had notably high OTA levels in maize, with an average of 181 µg/kg but ranging up to 350 µg/kg, (Z. U. Hassan et al. 2018), far above the European limit of 250 µg/kg for animal feed. In contrast, Poland reported much lower OTA contamination, with values from 0.13 to 86.0 µg/kg (Kosicki et al. 2016). This stark contrast further supports the observation that environmental and regional factors significantly influence mycotoxin contamination levels.

Among the countries presented in the literature, it is evident that China and Serbia are the ones with the highest overall levels of mycotoxin contamination, particularly with regard to DON and ZEN. Some of China’s DON levels in maize, reaching up to 19,811.0 µg/kg, were the highest reported for any mycotoxin across all regions. Similarly, a number of Serbia’s ZEN levels, particularly in maize, were alarmingly high, reaching a maximum of 9050.0 µg/kg.

On the other hand, regions such as Brazil and Norway, consistently exhibited lower mycotoxin contamination levels. For instance, in Brazil, the contamination levels of AFB_1_ in maize were substantially lower, with a maximum of only 6.5 µg/kg, and DON levels in maize were not excessively high. Similarly, Norway’s DON contamination in wheat remained very low, averaging just 28.3 µg/kg, and Poland’s OTA contamination, with a maximum of 86.0 µg/kg, remained well within safer limits, unlike Qatar’s levels.

The observed differences in mycotoxin contamination levels can likely be attributed to several factors, with weather conditions during crop growth and storage practices being the most influential. Mycotoxins, particularly those produced by fungi such as *Aspergillus, Fusarium,* and *Penicillium*, thrive in warm and humid conditions. This explains why countries with consistently hot and humid climates, such as parts of Southeast Asia and Kenya, tend to have higher levels of contamination. However, the relationship between climate and contamination is complex. For example, while some regions of China experience warm and humid conditions, much of its major grain production occurs in temperate zones with cold winters, which may limit fungal growth. Similarly, Serbia, with its moderate continental climate, experiences seasonal temperature variations that influence contamination levels differently than in tropical regions. An interesting exception to the general trend is Brazil, a developing country with a tropical climate that often reports relatively low mycotoxin contamination levels.

High humidity during the growing season and improper drying post-harvest techniques can encourage the growth of mycotoxin-producing fungi in crops like maize, wheat, and soybeans. This suggests that effective agricultural practices, regulatory measures, and post-harvest handling can play a crucial role in mitigating mycotoxin risks, even in high-risk climates. In contrast, regions with cooler and more temperate climates, such as Norway and Poland, tend to have lower mycotoxin contamination. Cooler temperatures and lower humidity levels during the growing season limit the development of mycotoxin-producing fungi. In addition, better storage practices, such as controlled temperature and humidity in warehouses, could further reduce the risk of fungal contamination during the storage period in those countries.

It is also important to emphasize the critical role of the post-harvest storage conditions in determining the levels of mycotoxins in feed ingredients. Poor storage practices, such as inadequate ventilation and high moisture levels in storage facilities, evidently lead to the growth of mold, which produces mycotoxins even after the crops have been harvested. This is particularly concerning in regions where proper storage infrastructure is lacking, which could explain the high mycotoxin levels in some developing countries, despite less favorable conditions for fungal growth in the field.

In conclusion, mycotoxin contamination of raw materials used in fish feed production is well-documented and widespread, but its occurrence varies across different regions and countries. While environmental factors during crop cultivation, such as temperature and humidity, play a key role in determining mycotoxin levels, storage practices are equally important. Both factors must be considered to understand and mitigate the risks of mycotoxin contamination in the feed ingredients. Therefore, addressing both agricultural and post-harvest practices is essential to ensure the safety and quality of raw materials used in fish feed production. However, it should be noted that feed producers often prescreen plant-derived ingredients, and processing steps, such as concentration or extrusion, can significantly reduce mycotoxin content before incorporation into commercial feeds. The reported concentrations illustrate the potential magnitude of contamination in plant-derived materials entering the aquaculture feed chain; however, their actual contribution to final feed composition depends on ingredient type, origin, and formulation practices, which may vary considerably among species and regions.

### Presence of mycotoxins in fish feed

With the increasing substitution of plant-based ingredients for fishmeal in aquaculture feeds, the potential risk of mycotoxin contamination is rising (Gonçalves et al. 2018a, b, c; Naehrer et al. 2012). However, detailed research on the contamination of commercially available fish feeds with multiple mycotoxins remains limited. Moreover, less is known about mycotoxin levels in fish feed than in plant-derived raw materials, making it difficult to assess potential consequences for aquaculture. Recent investigations, such as Søderstrøm et al. (2023), have highlighted the widespread occurrence of multiple mycotoxins in feed-related materials used in aquaculture and stressed the need for more comprehensive surveillance of compound feeds.

More recent studies indicate that contamination levels for some mycotoxins are substantially higher than those suggested by previous reports (Table 2). However, depending on the conditions and origin of the contamination, those levels can vary considerably (Pietsch 2020). The reported levels of mycotoxins in fish feed are generally lower than those reported in raw materials. This phenomenon could be simply explained by the “dilution effect” with the other feed components. However, pre-screening of plant-derived feed additives by feed producers and/or usage of high temperatures during feed extrusion process should also be considered as potential factors reducing the contents of FUMs, ZEN, DON, AFs and other mycotoxins (Bullerman and Bianchini 2007).

**Table 2:** Reported average levels of mycotoxins in commercially available fish feed

Mycotoxin	Material	Region	Content (μg/kg)	EU limit/recommendation*)	Reference
AFB_1_	Carp feed	Serbia	22	10 μg/kg	Agouz And Answer 2011
	Rainbow trout feed	Iran	0.12–20.9		Alinezhad et al. 2011
	Fish feed	Asia	52		Gonçalves et al. 2017
	Fish feed	Asia	52		Gonçalves et al. 2017
	Fish feed	Asia	173		Gonçalves et al. 2017
	Nile Tilapia, African catfish feed	Africa	71.0 ± 31.5		Marijani et al. 2017
	Fish feed	Brazil	0.1–16.5		Nogueira et al. 2020
DON	Fish feed	Asia	161	5000 μg/kg	Gonçalves et al. 2017
	Fish feed	Europe	166		Gonçalves et al. 2017
	Nile Tilapia, African catfish feed	Africa	245.8 ± 190.1		Marijani et al. 2017
	Fish feed	Southeast Asia	21.2		Gonçalves et al. 2018a, b, c
	Shrimp feed	Southeast Asia	121.5		Gonçalves et al. 2018a, b, c
FBs	Fish feed	Europe	3.420	10,000 μg/kg	Gonçalves et al. 2017
	Fish feed	Southeast Asia	88.0		Gonçalves et al. 2018a, b, c
	Shrimp feed	Southeast Asia	14.4		Gonçalves et al. 2018a, b, c
	Nile Tilapia, African catfish feed	Africa	1136.5 ± 717.9		Marijani et al. 2017
OTA	Fish feed	Brazil	2.2–31.6	250 μg/kg	Nogueira et al. 2020
ZEN	Fish feed	Asia	76.2	2000 μg/kg	Fegan And Spring 2007
	Rainbow trout feed	Poland	81.8 ± 25.8		Woźny et al. 2013
	Rainbow trout feed	Poland	10.3 ± 0.9		Woźny et al. 2013
	Fish feed	Central Europe	67.9		Pietsch et al. 2013
	Fish feed	Asia	60		Gonçalves et al. 2017
	Fish feed	Europe	118		Gonçalves et al. 2017
	Fish feed	Brazil	22.9–322.2		Nogueira et al. 2020

^*^EU limit/recommendation refers to guidance values for mycotoxins in feed for certain livestock. No official values exist specifically for fish; values shown are for feed materials commonly used in fish feed

The occurrence of mycotoxins in commercially available fish feed varies in both frequency and contamination levels across sampled regions. For example, the AFB_1_ contamination levels of fish feed in Asia reached as high as 173 μg/kg (Gonçalves et al., 2017a). This level of contamination was substantially higher than the European Union recommended limit of 10 μg/kg (EC, 2002). In contrast, OTA and FUMs were less frequently detected, with OTA levels ranging from 2.2 to 31.6 μg/kg in Brazilian fish feed (Nogueira et al. 2020), while FUM contamination was found to reach 3.42 μg/kg in European feed (Gonçalves et al., 2017b), well below the 10,000 μg/kg threshold. In turn, DON contents were some of the highest, particularly in Africa, where Nile tilapia and African catfish feed were contaminated with an average of 245.8 ± 190.1 μg/kg (Marijani et al. 2017). This however, was still well below the EU limit of 5,000 μg/kg (EC, 2006). Although ZEN contamination was widespread in both European and Asian samples, and its content was as high as 322.2 μg/kg in Brazilian fish feed, all these values were well below guidance levels (Fegan And Spring 2007; Gonçalves et al., 2017a; Nogueira et al. 2020; Woźny et al. 2013). Søderstrøm et al. (2023) further demonstrated that even feeds meeting regulatory guidelines can contain multiple co-occurring mycotoxins, including emerging compounds, which may act additively or synergistically to affect fish health.

When comparing contamination across regions, African and Asian countries displayed higher levels of mycotoxins in fish feed (Marijani et al. 2017; Gonçalves et al. 2018a, b, c). These trends are consistent with patterns observed in raw feed materials, where African and Asian regions tend to have higher contamination, likely due to climatic conditions favorable for mycotoxin production. Regional disparities in contamination levels for fish feed closely mirror those observed in other feed materials, without marked deviations suggesting specific regional vulnerabilities unique to finished feed.

Mycotoxins rarely occur individually in feed materials and complete feeds; more commonly, multiple compounds are present simultaneously. Among the most frequently identified mycotoxin mixtures were combinations of DON, ZEN, FUMs, and AFs (Gruber-Dorninger et al. 2018). Their detection, however, depends heavily on the methods and focus of the researchers. It is important to note that mycotoxins of most concern tend to receive significantly more attention than emerging mycotoxins. Information on the prevalence of the emerging mycotoxins is scarce, and in some cases, certain mycotoxins have not yet garnered sufficient attention from the scientific community.

In summary, several points deserve emphasis. First, mycotoxin contamination levels in fish feed are lower than in raw feed materials, particularly for AFs and FUMs. Second, with few exceptions, mycotoxin levels in fish feed remain below the regulatory guidelines, suggesting that these products are generally safe for aquaculture. However, importantly, research also indicates that even mycotoxins doses at or below the European Union’s guidance levels can have adverse effects, as detailed in the section below.

The potential presence of mycotoxins in fish meat and fish-derived products has raised growing concern in recent years. Although these are not primary targets of fungal contamination, mycotoxins can enter the aquatic food chain indirectly, primarily through contaminated feed in aquaculture or environmental conditions that promote fungal growth. While mycotoxins are rarely detected in fish and fish products, their presence is noticeable (Table 3).

**Table 3:** Occurrence of selected mycotoxins in fish and fish by-products

Mycotoxin	Source	Country/region	Content (μg/kg)	Reference
AFB_1_	Common carp raw meat	Northern Serbia	0.4–4.0	Rokvić et al. 2020
	Dried fish	China	0.03–3.52	Deng et al. 2020
	Dried seafood	China	0.58–0.87	Deng et al. 2020
	Dried fish	Chad	0.01–2.78	Ousman et. al 2019
AFB_2_	Dried fish	Chad	0.09–0.32	Ousman et. al 2019
AFs	Fish liver	Qatar	2.89 ± 1.37	Bashorun et al. 2023
	Fish raw meat	Qatar	1.38 ± 0.28	Bashorun et al. 2023
	Whole raw Tilapia	Egypt	0.55 ± 0.2	Anees et al. 2023
	Tilapia raw fillet	Egypt	0.68 ± 0.06	Anees et al. 2023
DON	Common carp raw meat	Northern Serbia	30.0	Rokvić et al. 2020
DON	Dried fish	China	0.71	Deng et al. 2020
ENA_1_	Sea bass raw meat	Europe	4.3	Tolosa et al. 2017
	Sea bream raw meat	Europe	4.0	Tolosa et al. 2017
	Atlantic salmon raw meat	Europe	25.5	Tolosa et al. 2017
	Rainbow trout	Europe	ND	Tolosa et al. 2017
ENB	Sea bass raw meat	Europe	12.8	Tolosa et al. 2017
	Sea bream raw meat	Europe	14.9	Tolosa et al. 2017
	Atlantic salmon raw meat	Europe	76.5	Tolosa et al. 2017
	Rainbow trout raw meat	Europe	3.6	Tolosa et al. 2017
ENB_1_	Sea bass raw meat	Europe	10.2	Tolosa et al. 2017
	Sea bream raw meat	Europe	12.7	Tolosa et al. 2017
	Atlantic salmon raw meat	Europe	75.0	Tolosa et al. 2017
	Rainbow trout raw meat	Europe	2.9	Tolosa et al. 2017
FB_1_	Common carp raw meat	Northern Serbia	40–4000.0	Rokvić et al. 2020
OTA	Common carp raw meat	Northern Serbia	1.6–16.0	Rokvić et al. 2020
	Fish liver	Qatar	1.30	Bashorun et al. 2023
	Fish raw meat	Qatar	ND	Bashorun et al. 2023
OTA	Whole raw Tilapia	Egypt	2.79 ± 0.6	Anees et al. 2023
	Tilapia raw fillet	Egypt	0.12 ± 0.01	Anees et al. 2023
	Dried fish	China	0.03–2.21	Deng et al. 2020
	Dried seafood	China	0.36–1.51	Deng et al. 2020
T-2	Common carp raw meat	Northern Serbia	10–100	Rokvić et al. 2020
	Dried fish	China	0.21–1.53	Deng et al. 2020
	Dried seafood	China	0.55–1.34	Deng et al. 2020
ZEN	Common carp raw meat	Northern Serbia	5–50.0	Rokvić et al. 2020
	Rainbow trout raw meat	Poland	ND	Woźny et al. 2013
	Rainbow trout raw meat	Poland	ND	Woźny et al. 2015
	Saltet roe of rainbow trout	Poland	< 5	Woźny et al. 2017
	Rainbow trout raw meat	Poland	ND	Woźny et al. 2019

ND—not detected

As mentioned in the previous sections, fish feed contamination with mycotoxins, such as AFs, OTA, DON, and FUMs, has been documented in various regions (Table 2). Fish exposed to mycotoxins through feed may accumulate these compounds in their tissues. However, several factors likely influence the extent of this mycotoxin accumulation, including the type of toxin, the level of feed contamination, environmental conditions, and the species of fish.

Certain mycotoxins have lipophilic properties, allowing them to persist in the fat tissues of animals that ingest them (Bozzo et al. 2023). Therefore, fat content in a fish may play a role, as concentrations of mycotoxins like ENNs tend to be higher in fatty tissues (Rodríguez-Carrasco et al. 2016). For example, in Atlantic salmon from European regions, ENB levels reached 76.5 µg/kg and those of ENB_1_ were as high as 75.0 µg/kg (Tolosa et al. 2017). These findings suggest that fish species with higher lipid content might be more susceptible to accumulating specific mycotoxins.

In turn, AFB_1_ accumulation reached 4.25 ± 0.85 μg/kg in the consumable muscle tissue of sea bass provided feed contaminated with this mycotoxin (El-Sayed et al. 2009). This finding raises concern about the potential transmission of AFB_1_ to human consumers, particularly given that the US Food and Drug Administration has established a 5 μg/kg limit for AFB_1_ in ready-to-eat food, compared to the European Union’s stricter limit of 2 μg/kg (EC 2006). Notably, AFB_1_ levels in dried fish and seafood from China ranged from 0.03 to 3.52 µg/kg (Deng et al. 2020), underscoring the importance of monitoring mycotoxin levels in these products. On the other hand, OTA levels in tilapia raw fillets were reported at 0.12 µg/kg (Anees et al. 2023). These contents, while concerning, would require substantial daily intake to surpass the recommended limits for mycotoxins. For context, the probability that fish intended for human consumption exceed European Union limits for contaminants like heavy metals (e.g. mercury or lead) in fish is higher. Heavy metals are more extensively studied and documented to accumulate in fish tissues compared to mycotoxins, as they persist in the environment, bioaccumulate, and increase in concentration through the food chain. In contrast, mycotoxins, while capable of entering aquatic systems through contaminated feed, tend to degrade more readily and do not typically undergo this progressive buildup, resulting in lower accumulation levels in fish tissues (Jia et al. 2017), and thus are subject to stricter regulatory limits (EFSA 2006).

Storage of fish-derived products under inadequate conditions may promote fungal growth and lead to further contamination. However, the methods of processing and storage that are used can also substantially affect the stability and content of mycotoxins in fish products. Drying, salting, and smoking are common preservation methods for fish, particularly in regions like China and Africa, helping to control fungal growth and mycotoxin production (Ousman et al., 2019; Deng et al. 2020). In a study, the effect of technological processing on the levels of ENNs has been examined, revealing that cooking methods commonly employed prior to fish consumption led to a reduction in mycotoxin levels. However, the mycotoxins were not entirely removed, as traces of ENB and ENB_1_ were identified in cooked samples at various contents. Moreover, new mycotoxin derivatives can be formed during thermal treatment of fish meat (Tolosa et al. 2017).

Mycotoxin levels usually reported in fish-derived products and other aquatic food are generally low. For example, in the food served at a university restaurant, DON was found in 17% of fish dishes and 36% of squid dishes at mean content of 1.19 to 3.58 μg/kg, whereas OTA was detected in 20% of prawn dishes with a mean level of 1.08 μg/kg (Carballo et al. 2018). Given contents like those reported in the study and a reasonable consumption of fish products for a typical consumer, it is unlikely that individuals would exceed the recommended daily intake of most mycotoxins (Leblanc et al. 2005). However, long-term risks to human health due to chronic exposure to low levels of mycotoxins, especially when combined with other dietary sources of contamination (particularly in developing nations or in areas where the diet includes high levels of cereals) cannot be neglected.

Research into mycotoxins in fish and fish products remains relatively underexplored compared to other food categories. While some studies have identified mycotoxins in various fish species, the extent of contamination, regional differences, and species-specific susceptibilities require further investigation. A significant limitation of studies to date is due to the lack of large-scale, systematic monitoring programs for mycotoxins in aquatic systems. Moreover, the focus on certain mycotoxins, such as AFs, means that there exists a need for research on less-studied toxins like ENNs, which were found at relatively high concentrations in European fish (Tolosa et al. 2017).

Future studies should emphasize the role of aquaculture practices in preventing mycotoxin contamination, as well as the potential for climate change to alter fungal growth patterns and mycotoxin distribution. Another key area of research is the development of effective processing methods to reduce mycotoxin levels in fish products without compromising nutritional value. Additionally, more systematic research on fish metabolism of mycotoxins and deeper understanding of the synergistic effects of these compounds with other contaminants, such as heavy metals, is necessary to accurately assess the risk to human health.

In conclusion, while mycotoxins are present in fish and fish products at varying levels, the contents typically observed do not pose a significant risk to consumers. However, ongoing research and monitoring are essential to ensure the continued safety of these products in the food chain.

## Effects of mycotoxins on aquaculture fish species

Although most scientific studies on toxicity of mycotoxins have focused on a few widely farmed animals (i.e. pigs, cattle, or poultry), research on the effects of mycotoxins in fish has intensified over the past decade. The relevant studies have involved numerous species popular in aquaculture such as carp, catfish, trout, or tilapia (Gonçalves, et al. 2018a, b, c; Oliviera And Vasconcelos 2020). From impaired growth and increased susceptibility to pathogen infections, to cellular and subcellular compartment damage, as well as hormone-mimicking actions, mycotoxins exhibit a broad range of effects that depend primarily on their chemical structure and dose. In this section, we focus on the known biological properties of mycotoxins in fish, limiting the discussion to those mycotoxins that are known to have the greatest impact on aquaculture.

### Zearalenone

The toxicokinetic and toxicodynamic details of ZEN have been studied in detail in only a few farm animal species (mainly mammals and birds). Shortly after ingestion of contaminated feed, ZEN is rapidly absorbed from the gastrointestinal tract and metabolized in the liver and intestine to its two major metabolites, α- and β-zearalenol. These metabolites are then reduced to α- and β-zearalanol (also referred to as zeranol and taleranol, respectively). In the next phase of metabolism, these compounds are conjugated, mainly with glucuronic acid, and then excreted via bile or urine (Dänicke and Winkler 2015; Ropejko and Twarużek, 2021). Although studies on the metabolism of this mycotoxin in fish strongly suggest that the biotransformation pathways are similar to those in mammals or birds (Laganà et al. 2004; Malekinejad and Agh 2016; Woźny et al. 2017), there is still a knowledge gap regarding the specific activity (biological properties) of the metabolites formed by fish in response to the presence of the parent compound in contaminated feed. Research on fish has mainly described the effects of exposure to ZEN in water on a fish species serving as a laboratory model. Importantly, however, the potential for ZEN-contaminated feed to affect fish health and reproduction has also been described for commercially important fish species, like rainbow trout or grass carp (Table 4).

**Table 4:** Effects of zearalenone on aquaculture fish species

Fish species	Dose	Exposure period	Physiological effect	Biochemical effect	Reference
*Acipenser dabryanus*	1030 μg/kg feed	7 weeks (oral exposure)	decreased serum levels of TP, albumin, TGs, total cholesterol and low-density lipoprotein cholesterol; altered gut microbiota diversity	NM	Wu et al. 2021
*Cyprinus carpio*	332–797 μg/kg feed	4 weeks (oral exposure)	increased oxygen consumption; decreased serum TP content	decreased LDH activity	Pietsch and Junge 2016
*Ctenopharyngodon idella*	500–2500 μg/kg feed	70 days (oral exposure)	increased morbidity; intensified effects of *A. hydrophila* infection (intestinal swelling and redness)	decreased immune component activities (ACP, LZ, C3, C4) and IgM content in foregut, midgut and hindgut	Zhang et al. 2021
*Ctenopharyngodon idella*	535–2507 μg/kg feed	70 days (oral exposure)	impaired gill epithelial barrier function due to differential expression of TJs; increased oxidative stress levels, excessive apoptosis	decreased immune component activities (ACP, LZ, C3, C4) and AMPs levels	Zhang et al. 2023
*Danio rerio*	100–3200 ng/l	182 days (waterborne exposure)	decreased fecundity; feminizing effect; decreased SF and clutch size	increased levels of vtg;	Schwartz et al. 2010
*Danio rerio*	0.1–1000 µg/L	21 days (waterborne exposure)	upward curvature of the body axis	increased mRNA and protein levels of vtg	Bakos et. Al 2013
*Danio rerio*	350–950 μg/L	24, 48, 72 and 96 hpf (waterborne exposure)	developmental defects (pericardial edema, hyperemia, yolk sac edema, spine curvature), reduction in heart rate	reduction in antioxidant defense system (SOD, CAT, GPx, GST and GSH) and changes in metabolic biomarkers (LDH and AP); inhibition of AChE activity in higher exposure groups	Muthulakshmi et. Al 2018
*Danio rerio* *Paralichthys olivaceus* *Ocyurus chrysurus*	0.5 to 5 ppm or 0.5 to 100 ppm	24 h (waterborne exposure)	membrane disruption, mitochondrial dysfunction	increased production of ROS; impaired energy metabolism	Annunziato et. Al 2023
*Dicentrarchus labrax*	0.725 g/kg feed	4 weeks (oral exposure)	inhibited fish growth and feed utilization; decreased RBCs, Hb, Ht, and MCHC; increased MCV and MCH; decreased serum levels of TP, globulin, and LZ	decreased activity of SOD, CAT, and GPx; decrease in mRNA expression of IL-4 and IL-1β genes; increased transcription of TNF-α and HSP70 genes in the liver and kidney	Abdel-Tawwab et. Al 2021
*Oncorhynchus mykiss*	10 mg/kg body mass	7 days (intraperitoneal injection)	interference with blood coagulation, iron storage	decreased levels of Fe in the liver, increased levels of blood clotting time	Woźny et al. 2012
*Oncorhynchus mykiss*	1.81 mg/kg feed	71 days (oral exposure)	histopathological alterations in the liver (necrotic areas, disorders of polygonal hepatocytes, cytoplasm vacuolization, and macrophage aggregates); more advanced ovarian development in females	statistically unclear exposure effect on vtg mRNA level in the liver of females	Woźny et al. 2015
*Oncorhynchus mykiss*	2040.6 µg/kg feed	96 weeks (oral exposure)	increased growth rate and feeding efficiency; macroscopic changes in trunk-kidney caused by *Tetracapsuloides bryosalmonae* infection; decreased number of B lymphocytes, increased number of thrombocytes; altered A:G in blood	alterations in expression of cytokine proteins (IL-4, IL-12, IL-17, and IFNγ) in the immune-related organs	Woźny et al. 2019
*Oncorhynchus mykiss*	2040.6 μg/kg feed	96 weeks (oral exposure)	interference with sex differentiation; increased sperm concentration in males; increased risk of offspring death	increased protein levels of vtg in the blood of males	Woźny et al. 2020

At the molecular level, ZEN affects the reproductive system of fish by mimicking the action of estradiol, an important sex hormone that regulates the development and function of the reproductive system (Ropejko and Twarużek 2021). After passive entry through the cell membrane, this mycotoxin binds to specific nuclear receptors, i.e. the estrogen receptors (ERs). All ZEN derivatives exhibit some estrogenic property, but it is the reduced form, α-zearalenol, that has a stronger estrogenic potency than other ZEN metabolites (Grgic et al. 2022; Ropejko and Twarużek 2021). Following activation by ligand binding, the ERs trigger a series of molecular responses (e.g. increased expression of vitellogenin, an egg yolk protein, in the liver) that drive sexual maturation in fish (Arukwe and Goksøyr 2003).

Due to natural differences in hormone levels and physiological responses, the effect of ZEN on the reproductive health of fish varies greatly depending on the sex of the exposed individual. In male fish, ZEN exposure (especially in early developmental stages) can lead to feminization, impair sex differentiation, reduce sperm production and alter testicular development. Although female fish appear to be less susceptible, prolonged exposure to ZEN or exposure to higher doses can lead to disturbances in ovarian development, resulting in reduced egg quality and fertility (Schwartz et al. 2010; Woźny et al. 2013). In the affected farmed fish species, the imbalance of sex hormones caused by ZEN can ultimately lead to impaired reproductive success and a decline in the population causing losses in livestock production (Woźny et al. 2020).

As steroids are generally considered to be strong regulators of the immune system (Mokhtar et al. 2023), the hormone-mimicking activity of ZEN has also been linked to its negative effects on the immune system of exposed fish. Exposure of fish to ZEN in feed can effectively suppress the activity of components of the adaptive and innate immune system, thus increasing individual susceptibility to pathogen infections and diseases. Long-term dietary exposure of rainbow trout to ZEN at a dose of 2 mg/kg of feed, a value aligned with the EC guidance limit for feed ingredients (not compound feed), resulted in marked changes in the blood leukogram and disruption of cytokine expression in major organs (Woźny et al. 2019). The head kidneys of fish juveniles exposed to ZEN in feed for 72 weeks showed massive inflammation caused by infection with *Tetracapsuloides bryosalmonae*, strongly suggesting that the exposure may have impaired the immune functions of the fish (Woźny et al. 2019). In another comprehensive study on grass carp, fish fed with ZEN-contaminated feed at doses between 1 and 2.5 mg/kg feed for two months showed a higher incidence of enteritis and an increased enteritis morbidity rate after infection with *Aeromonas hydrophila*, which was used as an experimental model to induce intestinal inflammation (Zhang et al. 2021). Higher susceptibility to infection with the pathogen was associated not only with the dose-dependent content of ZEN in the gut of the exposed fish, but also with a functional impairment of various compartments of the digestive tract immune system (Zhang et al. 2021). In a follow-up study with the same fish species (grass carp) and the same exposure regime (two months of feed-borne exposure), the fish exposed to ZEN at doses between 1 and 2.5 mg/kg feed showed a higher incidence of gill rot following infection with *Flavobacterium columnare* (Zhang et al. 2024a, b). The infection symptoms were associated with a decrease in the activity of selected immune components (acid phosphatase, lysozyme, complement component 3) and antimicrobial peptides (AMPs), and a significantly increased concentration of inflammatory cytokines in the gills (Zhang et al. 2024a, b).

In addition to the reported reproductive and immune system disorders, studies on fish exposure to ZEN have also demonstrated other various toxic effects, including developmental defects in fish embryos, histopathological changes in vital organs, altered enzyme activities, and disruption of metabolic processes leading to abnormal growth rates or even behavioral changes. Most of these toxicological studies point to oxidative stress as a general mechanism of the toxic effect of ZEN (Table 4). It is suggested that exposure of fish to ZEN results in an accumulation of reactive oxygen species (ROS) and a reduction in the activity of antioxidant enzymes, which in turn leads to oxidative damage. For example, in a study on zebrafish embryos, a significant induction of oxidative stress markers (ROS, LPO and NO) and a reduction in elements of the antioxidant defense system (SOD, CAT, GPx, GST and GSH) was observed, indicating that oxidative stress is a major factor in ZEN-induced toxicity (Muthulakshmi et al. 2018). In a recent study that employed metabolic profiling of fish embryos exposed to sub-lethal concentrations of ZEN, metabolites that are mainly associated with oxidative stress, membrane disruption, mitochondrial dysfunction and impaired energy metabolism were identified. That study also revealed a large overlap between the biological responses of marine fish embryos and those of freshwater fish embryos (olive flounder, yellowtail snapper and zebrafish). Those results were supported by an increased tissue-specific production of ROS, also indicating that oxidative stress is a major mechanism of ZEN toxicity (Annunziato et al. 2023).

### Aflatoxin B1

In fish, aflatoxin B1 (AFB_1_) exposure leads to a range of physiological disruptions (Table 5). AFB_1_ mainly impairs hepatic function in fish, resulting in decreased detoxification capacity, increased oxidative stress, and alterations in carbohydrate and lipid metabolite mobilization (Barany et al. 2021). In common carp (*Cyprinus carpio*), exposure to this mycotoxin leads to hepatic lesions, including large areas of necrosis and fatty degeneration (Sherif et al. 2021; Tasa et al. 2020; Vaziriyan et al. 2018). Furthermore, in Nile tilapia *(Oreochromis niloticus*) and grass carp (*Ctenopharyngodon idella*), histopathological changes in the gills, liver, and kidneys occur, also demonstrating the multi-organ toxicity of AFB_1_ (Anh Tuan et al. 2002; Arana et al. 2014; Chávez-Sánchez et al. 1994; Deng et al. 2020; Khan et al. 2019; Zeng et al. 2019; Zychowski et al. 2013b). Moreover, exposure to AFB_1_ is associated with decreases in the hepato-somatic index (HSI), an important indicator of fish health status, as well as higher levels of liver enzymes like AST, ALT, and ALP (Gonçalves et al. 2018a, b, c; Vaziriyan et al. 2018; Khan et al. 2019; Li et al. 2022; Sherif et al. 2021; Peng et al. 2021). At the molecular level, AFB_1_ up-regulates *Keap1a* expression, which suppresses *Nrf2* signaling and decreases both mRNA levels and the activities of antioxidant enzymes, ultimately leading to elevated ROS levels in the liver. These changes are further indicators of AFB_1_-induced hepatotoxicity in fish (Zeng et al. 2019; Liu et al. 2023).

**Table 5:** Effects of aflatoxin B1 on aquaculture fish species

Fish species	Dose	Exposure period	Physiological effect	Biochemical effect	Reference
*Acipenser barei* ♀ × *Acipenser ruthenus* ♂	1–80 μg/kg feed	35 days (oral exposure)	increased mortality and Ht; nuclear hypertrophy, hyperchromasia, extensive biliary hyperplasia, focal hepatocyte necrosis, presence of inflammatory cells in the liver	NM	Rajeev Raghavan et al. 2011
*Carassius gibelio*	3.3–1646.5 μg/kg feed	24 weeks (oral exposure)	decreased GSI, brood amount (fecundity), and oocyte diameter; AFB_1_ accumulation in tissue of ovaries and muscles	statistically unclear exposure effect on the activity of ALT, AST	Huang et al. 2014
*Carassius auratus gibelio*	50–100 µg/kg feed	28 days (oral exposure)	increased mortality; higher viral load in the fish liver, kidney, and spleen	induced oxidative stress; increased ROS and MDA levels; reduced activity of SOD, GST and CAT; down-regulated expression of Nrf2, IRF3, and IFN1	Xue et al. 2023
*Channa argus*	50–400 μg/kg feed	56 days (oral exposure)	damage and lesions in the liver	increased levels of AST, ALT, LDH, ALP	Li et al. 2022
*Ctenopharyngodon idella*	29–147 μg/kg feed	60 days (oral exposure)	decreased growth performance and deformities in juveniles, histopathological alterations in the head kidney and spleen	attenuated antioxidant ability through up-regulation of *Keap1a* and suppression of Nrf2 signaling, leading to decreased mRNA levels and activities of antioxidant enzymes; apoptosis increased via *p38 MAPK* activation	Zeng et al. 2019
*Ctenopharyngodon idella*	25–100 µg/kg feed	49 days (oral exposure)	lowered RBC, WBC, Ht, Hb, MCV, lymphocytes count in the blood; pathological changes in the liver, kidney, intestine and gills	increased serum levels of AST, ALT, glucose, urea, and creatinine	Khan et al. 2019
*Ctenopharyngodon idella*	30 – mg/kg feed	28 days (oral exposure) and 14 days of exposure with *A. hydrophila* challenge	reduced antibacterial compounds and immunoglobulin contents; decreased transcription level of AMPs in immune organs	increased transcription level of pro-inflammatory cytokines and decreased transcription level of anti-inflammatory cytokines in immune organs, which might be regulated by NF-kB and TOR signaling	He et al. 2022
*Cyprinus carpio*	0.5–1.4 mg/kg feed	3 weeks (oral exposure)	changes in plasma biochemical indices; internal bleeding, liver damage, pale gills	decreased levels of serum ALP activity, TP, and globulin; increased plasma AST and LDH activities; increased glucose, cholesterol, TGs, and creatinine	Vaziriyan et al. 2018
*Cyprinus carpio*	50–400 µg/kg feed	12 weeks (oral exposure)	lesions in the intestine, including necrosis, immune cell infiltration, and fibroplasia corresponded to increase in AFB_1_ diet levels	increased in activity of intestinal alkaline protease, lipase, and amylase corresponded to increase in AFB_1_ diet levels	Tasa et al. 2020
*Cyprinus carpio*	0.5–1.0 mg/kg feed	14 days (oral exposure)	degenerative changes in hepatic and spleen tissues, loss of appetite, lethargy, loss of reflexes at the end of the experiment	increased activity of serum AST, ALT, and ALP, decreased levels of IL-1β and TNF-α mRNA in the head kidney	Sherif et al. 2021
*Danio rerio* (embryos)	0.25–0.5 μM	96 hpf (waterborne exposure)	reduced liver size, disrupted hepatocyte structure	decreased expression of genes engaged in embryonic development (*Hhex* and *Prox1*); expression activation of genes involved in controlling cellular stress and damage (*tp53*, *mdm2*, *puma*, *noxa*, *pidd1*, and *gadd45aa)*; activation of genes that play a role in apoptosis (*baxa*, *casp8*, and *casp3a*)	Cheng et al. 2021
*Danio rerio* (embryos)	0.03125–0.5 mg/L	4 to 120 hpf (waterborne exposure)	increased neutrophil granulocyte influx, nitric oxide production, and yolk lipid accumulation; defective gastrointestinal tract development; reduced L-arginine levels	increased expression of inflammatory gene network; repression of lipid metabolism and gastrointestinal tract development-related gene sets	Ivanovicset al. 2021
*Dicentrarchus labrax L*	0.018 mg/kg body weight	42 days (oral exposure)	darkening of body surface, hemorrhagic and yellowish patches on the dorsal skin surface; eye opacity; pale discoloration of the gills, liver, and kidney; severe distension of the gall bladder	increase in serum transaminases and ALP; decrease in plasma proteins	El-Sayed And Khalil 2009
*Epinephelus fuscoguttatus* ♀ × *Epinephelus lanceolatus* ♂	7–2230 μg/kg feed	8 weeks (oral exposure)	decreased growth performance; liver damage, hepatic inflammation; increased liver ROS levels	increased activity of genes involved in energy regulation (*ampk*), fat breakdown (*hsl*) and protection of cells from stress (*keap1*); decreased activity of genes related to fat production and storage (*srebp1*, *ppar-γ*), inflammation (*tnf-α*, *inf-α*), and fatty acid absorption (*lfabp*)	Liu et al. 2023
*Lateolabrax maculatus*	0.1–1.0 mg/kg feed	56 days (oral exposure)	decreased growth performance, HSI, VSI, ISI	decreased serum levels of albumin, high density lipoprotein cholesterol, and glucose; modulated levels of AST; increased serum levels of TAOC, SOD, MDA, ALP, and LZM; increased immunoglobulin M in the liver	Peng et al. 2022
*Oncorhynchus mykiss*	0.5 ppm	24h passive egg/embryo exposure by bath treatment	mixed hepatocellular/cholangiocellular carcinoma	formation of DNA adducts	Bailey et al. 1994
*Oncorhynchus mykiss*	80 μg/kg feed	12 months (oral exposure)	hepatocytes displayed eosinophilia and an increase in pyknotic nuclei; large and eosinophilic hepatocytes with karyomegaly or binucleation; large areas of necrosis in liver; oval cell proliferation	NM	Arana et al. 2014
*Oreochromis niloticus*	0.94–3.0 mg/kg feed	25 days (oral exposure)	decreased growth rate and feed consumption; fatty liver, neoplastic changes (nuclear and cellular hypertrophy, nuclear atrophy, increase in number of nucleoli, cellular infiltration, hyperemia, cellular basophilia) and necrosis; congestion and shrinking of the glomeruli; melanosis in the kidney	NM	Chávez-Sánchez et al. 1994
*Oreochromis niloticus*	0.25–100 μg/kg feed	8 weeks (oral exposure)	reduced weight gain and Ht, liver contained excess lipofuscin and irregularly sized hepatocellular nuclei; weight loss and severe hepatic necrosis; increased mortality	NM	Tuan et al. 2002
*Oreochromis niloticus*	0.350–1.177 mg/kg feed	30 days (oral exposure)	synergistic action of AFs and *Aeromonas hydrophila* affecting feed conversion and the length of the fish	NM	Oliveira et al 2013
*Oreochromis niloticus*	1.5–3.0 ppm feed dry weight	10 weeks (oral exposure)	decreased weight gain, feed efficiency, HSI and macrophages; hepatocyte necrosis, cellular pleomorphism, nuclear pleomorphism, dysplasia, hydropic degeneration, and fatty degeneration of the liver	AFB_1_ binding to DNA; formation of AFB_1_−8,9-epoxide, involved in the development of fatty liver, necrosis and carcinogenesis; decreased extracellular superoxide anion production	Zychowski et al. 2013b
*Oreochromis niloticus*	3.2–16.2 mg/kg feed	20 days (oral exposure)	decreased weight gain, liver damage at higher dose	induction of biotransformation enzymes (EROD, GST, UGT, and SULT) involved in detoxification in the liver	Deng et al. 2020
*Oreochromis niloticus*	0–3 ppm	84 days (oral exposure)	reduced growth, feed utilization	decreased activity of digestive and antioxidant enzymes; increased activity of liver enzymes; DNA damage; increased secretion of endogenous amylase and chymotrypsin	Hassan et al. 2020
*Oreochromis niloticus* × *O. aureus*	19–1641 μg/kg feed	20 weeks (oral exposure)	reduced growth; induction of hepatic disorders, resulting in decreased lipid content and HSI	increased cytochrome P450 A1 activity, elevated plasma ALT activity	Deng et al. 2010
*Pangasius hypophthalmus*	50–1000 μg/kg feed	12 weeks (oral exposure)	decreased weight gain, increased HSI, ASI; liver damage; compromised disease resistance	increased levels of serum ALT and AST	Gonçalves et al. 2018a, b, c
*Pelteobagrus fulvidraco*	200–1,000 μg/kg feed	12 weeks (oral exposure)	decreased survival rate; reduced weight gain, final body weight, and SGR; increased FCR; reduced serum levels of TP; increased A:G	decreased bactericidal activity and LZM activity of the serum	Wang et al. 2016
*Pelteobagrus fulvidraco*	44–234 μg/kg feed	8 weeks (oral exposure)	increased total lipid and TG contents in muscle; decreased moisture content in flesh, TP, and PL; reduced myofiber diameter	decreased expression of genes related to myofiber structural proteins with increased dietary AFB_1_ content	Zhang et al. 2021
*Rhamdia quelen*	1177 μg/kg feed	21 days (oral exposure)	increased locomotor activity	increased activity of AChE brain synaptosomes; decreased activity of the sodium–potassium pump	Baldissera et al. 2018
*Rhamdia quelen*	45–180 μg/kg feed	56 days (oral exposure)	hematological and biochemical changes consistent with metabolic disorders in liver, gill, and renal tissue	increased serum ALP levels	Anater et al. 2020
*Sciaenops ocellatus*	0.1–5 ppm	7 weeks (oral exposure)	reduced weight gain, survival, and feed efficiency	reduced serum LZ concentration, HSI, whole-body lipid levels, liver histopathological scoring, and trypsin inhibition	Zychowski et al. 2013a
*Sparus aurata*	2 mg/kg feed	85 days (oral exposure)	altered mobilization of carbohydrate and lipid metabolites	increased expression of genes involved in steroidogenesis and the growth hormone signaling pathway	Barany et al. 2021
*Sparus aurata*	1–2 mg/kg feed	85 days (oral exposure)	impaired growth, tissue integrity, altered metabolism, and reduced TW; depleted levels of carbohydrate and lipid metabolites in plasma and liver	reversed relationship between pituitary growth hormone (*gh*) and liver hormone (*igf1*); increased activity of genes related to stress (*trh*, *crh*, *crhbp*) in the hypothalamus and of a steroid production gene (*star*) in the head kidney	Barany et al. 2021

Importantly, Eurasian carp (*Cyprinus carpio*), Yellowhead catfish (*Pelteobagrus fulvidraco*), and Asian seabass (*Lateolabrax maculatus*) fed with a diet containing AFB_1_ show decreased levels of TP, albumin, globulin, and HDL (Wang et al. 2016; Vaziriyan et al. 2018; Peng et al. 2022). As a result of these adverse effects, fish display poor growth performance, loss of appetite, reduced liver size, and lower final body weight (Chávez-Sánchez et al. 1994; Deng et al. 2010; Zychowski et al. 2013b; Wang et al. 2016; Gonçalves et al. 2018a, b, c; Zeng et al. 2019; Deng et al. 2020; Hassan et al. 2020; Barany et al. 2021; Cheng et al. 2021; Peng et al. 2021; Sherif et al. 2021).

The main molecular mechanism underlying AFB_1_'s hepatotoxic effects on fish involves its binding to DNA, resulting in the formation of AFB_1_−8,9-epoxide, which contributes to the onset of fatty liver, necrosis, and carcinogenesis (Klein et al. 2000). This has been observed in rainbow trout developing hepatocellular carcinoma after exposure (Bailey et al. 1994).

AFB_1_ also exhibits immunomodulatory effects on both the innate and adaptive immune components. In AFB_1_-exposed fish, mortality rates are higher and immune responses are compromised (Rajeev Raghavan et al. 2011; X. Wang et al., 2016b; Xue et al. 2023). Exposed fish are less able to mount an effective defense against pathogens, making them more susceptible to opportunistic infections, and they display alterations in leukocyte counts and impaired phagocytic activity (Chávez-Sánchez et al. 1994; Zychowski et al. 2013b; He et al. 2022). Furthermore, AFB_1_ exposure is linked to disruptions in cytokine regulation, influencing the inflammatory response in fish. Similarly in common carp (*Cyprinus carpio*) and Nile tilapia (*Oreochromis niloticus*), pro-inflammatory cytokines are upregulated, and anti-inflammatory cytokines are downregulated after AFB_1_ exposure (Arana et al. 2014; Khan et al. 2019).

The adverse effects of AFB_1_ on the fish immune system extend to the modulation of immune cell functions. AFB_1_ exposure has been associated with alterations in lymphocyte proliferation and activity, affecting the adaptive immune response (Tuan et al. 2002; Zeng et al. 2019). Changes in the expression of immune-related genes reflect the molecular mechanisms underlying AFB_1_-induced immunotoxicity in fish (Ivanovics et al. 2021; Cheng et al. 2021). AFB_1_ affects gene expression in zebrafish (*Danio rerio*) embryos, upregulating genes associated with inflammation and downregulating genes involved in antioxidant defense. These findings demonstrate that at the molecular level in fish, AFB_1_ is able to induce oxidative stress and numerous inflammatory responses (Cheng et al. 2021; Ivanovics et al. 2021).

In summary, the evidence available in the literature clearly shows the issue of AFB_1_ contamination in fish feed and aquaculture, as this mycotoxin poses a significant threat to fish health by inducing physiological, histological, and molecular alterations that ultimately impair growth performance.

### Deoxynivalenol

Deoxynivalenol (DON), a common mycotoxin produced by various *Fusarium* species, poses a significant threat to aquatic organisms, particularly fish. Extensive research has documented the diverse physiological, histological, and molecular effects of DON on multiple fish species, highlighting its detrimental impact on aquaculture systems (Table 6).

**Table 6:** Effects of deoxynivalenol on aquaculture fish species

Fish species	Dose	Exposure period	Physiological effect	Biochemical effect	Reference
*Salmo salar*	0.5–6.0 mg/kg feed	8 weeks (oral exposure)	reduced FI, weight gain, body length; increased liver weight	decreased levels of blood ALKP, cholesterol, TG, TP and albumin	Bernhoft et al. 2018
*Oncorhynchus mykiss* and *Oreochromis niloticus*	0.1–1.3 ppm	10 weeks (oral exposure)	decreased weight gain, TGC, feed efficiency, whole body CP content, RN	activation of MAPKs	Hooft et al. 2019
*Oncorhynchus mykiss*	< 0.5–6 ppm	7 weeks (oral exposure)	reduced FI; decreased mortality after infection with *Flavobacterium psychrophilum*	NM	Ryerse et al. 2016
*Oncorhynchus mykiss*	46–1300 μg/kg feed	8 weeks (oral exposure)	decreased growth rate; increased FCR; decreased protein retention efficiency; supranuclear vacuoles in the midgut area	NM	Koletsi et al. 2023
*Ctenopharyngodon idella*	27–1515 μg/kg feed	60 days (oral exposure)	increased enteritis morbidity after challenge with *A. hydrophila*	decreased activity of LZ and ACP, C3, C4 and IgM; down-regulation of immune system-related genes (LEAP −2A, LEAP-2B, hepcidin, β-defensin-1 and mucin2); up-regulation of mRNA expression of cytokines IL-1β,TNF-α, and INF-γ2	Huang et al. 2019
*Ctenopharyngodon idella*	27–1515 μg/kg feed	60 days (oral exposure)	body malformation; histopathological lesions, oxidative damage, declining antioxidant capacity, apoptosis, and destruction of TJs in the intestine	increased oxidative stress (increase in levels of ROS MDA, PC), reduced antioxidant defenses (reduced levels of GSH), cell death signals (increased levels of caspases 3, 7, 8, 9, Apaf-1, Bax, FasL, and NK), and changes in tight junction proteins (reduced expression of ZO-1, ZO-2b, occludin, and increased expression of claudin-12, claudin-15a, and MLCK)	Huang et al. 2018
*Salmo salar*	5.5 mg/kg feed	8 weeks (oral exposure)	no histopathological changes in the intestines	decreased expression of markers for tight junction proteins (claudin 25b, occludin, and tricellulin), increased expression of a marker for proliferating cell nuclear antigen	Moldal et al. 2018
*Oncorhynchus mykiss*	1,166–2,745 μg/kg feed *367 μg/kg feed	50 days *168 days (oral exposure)	reduced growth performance; mild to moderate changes in the liver; reduced FI and final weight; abnormal body shapes	increased activity of the liver enzymes (ALT and AST) in the blood	Gonçalves et al. 2019
*Scophthalmus maximus*	3 mg/kg feed	67 days (oral exposure)	decreased weight gain, SGR, and feed efficiency ratio	reduced content of immune system compartments (IgM and C4) in serum; decreased activity of SOD, CAT and TAOC, but increased MDA content	Wang et. Al 2021
*Oncorhynchus mykiss*	4.5–10.5 mg/kg feed	60 days (oral exposure)	increased mortality; decreased FI, FBW, and SGR	up-regulated expression of genes involved in stress response, appetite regulation, and energy balance (increased levels of *npy* and *adcyap1a/PACAP* mRNAs, and of the enzyme precursor *try3*)	Gonçalves et al. 2018a, b, c
*Cyprinus carpio*	1.75 mg/kg body weight	24h (oral exposure)	rapid and marked response of antioxidant defense to ROS formation	increased levels of GSH content and GSH-Px activity; expression modulation of genes involved in oxidative stress response (*gpxa, gpx4a, nrf2*)	Kövesi et al. 2020
*Oncorhynchus mykiss*	0.7–2.1 ppm 2.1–5.9 ppm	8 weeks (oral exposure)	decreased weight gain and feed efficiency; decreased TGC, FI, whole body CP content, RN, RE and NRE; increased number of apoptotic/necrotic cells in the pyloric caeca and decreased number of mitotic cells in the pyloric caeca and liver	activation of MAPK signaling pathway	Hooft et al. 2019
*Ctenopharyngodan* *idella*	27–1515 μg/kg feed	60 days (oral exposure)	oxidative injury; apoptosis and disruption of TJs in the gills	expression modulation of Nrf2, JNK, and MLCK signaling pathways, involved in stress responses, cell death, and cell function	Huang et al. 2020
*Oncorhynchus mykiss*	< 100–1600 μg/kg feed	8 weeks (oral exposure)	hemorrhages and histopathological changes in the liver; mild damage in the gastrointestinal tract	down-regulation of interleukins (IL-1β, IL-8, TNF-α)	Koletsi et al. 2022
*Cyprinus carpio*	953 μg/kg feed	56 days (oral exposure)	affected proportions of leukocyte cell types (lymphocytes, thrombocytes, monocytes, and granulocytes)	modulation of mRNA levels of immune genes in head kidney, trunk kidney, spleen, liver, and intestine (activation of both pro- and anti-inflammatory cytokines)	Pietsch et a. 2015
*Oncorhynchus mykiss*	0.3–2.0 ppm	12 weeks (oral exposure)	decreased weight gain, FI and TGC, as well as whole body CP, lipid, ash and gross energy content; increased whole body water content	NM	Hooft and Bureau 2017

DON exposure in fish leads to a range of physiological disruptions, affecting growth performance and overall health. The most commonly observed effect of DON exposure in fish is reduced feed intake, leading to subsequent growth suppression. This effect is thought to be mainly due to the anorectic (appetite-suppressing) action of DON, which is mediated via different mechanisms in the brain and the gastrointestinal system (Gonçalves et al. 2019; Koletsi et al. 2023). For example, exposure to dietary DON compromises growth performance and selected health indices in Atlantic salmon (*Salmo salar*) (Bernhoft et al. 2018). Similarly, rainbow trout (*Oncorhynchus mykiss*) exposed to DON-contaminated feed exhibit impaired growth performance, nutrient utilization, and histopathological alterations (Hooft et al. 2019), and the severity of these physiological effects is influenced by the content of DON and the duration of exposure (Gonçalves et al. 2019).

DON may compromise the integrity of the intestinal epithelial barrier and modulate the inflammatory response (cytokine signaling), as indicated by histological examinations of Atlantic salmon (Moldal et al. 2018). Similarly, grass carp (*Ctenopharyngodon idella*) exposed to DON exhibit structural alterations in the intestines and gills, including decreased immune function, oxidative damage, apoptosis, and disruption of tight junctions (Huang et al. 2018, 2019). These histopathological changes not only highlight the vulnerability of gut tissues of fish to DON toxicity, but also demonstrate that this mycotoxin is a modulator of the immune system in fish (Pietsch et al. 2014; Huang et al. 2019).

At the molecular level, DON disrupts eukaryotic protein synthesis by binding to ribosomes, which leads to a ribotoxic stress response that activates mitogen-activated protein kinases (MAPKs). This effect can result in either immune compartments activation or suppression, observed as upregulation of cytokines, chemokines, and other proinflammatory-related proteins, or in cases of longer exposure to higher doses, apoptosis and cell cycle arrest, as well as immunosuppression (Hooft and Bureau 2021). DON also exerts profound effects on gene expression and various signaling pathways in fish, such as downregulation of the NF-κB and TOR signaling pathways in grass carp, compromising immune function and growth regulation (Huang et al. 2018, 2019). Similarly, the CYP450s/ROS/PI3K/AKT pathway is involved in DON-induced apoptosis and necroptosis in carp neutrophils (Ding et al. 2021). Following exposure of common carp (*Cyprinus carpio*) to DON, regulatory gene expression is altered, as well as the lipid peroxidation and the glutathione redox systems (Kövesi et al. 2020).

The cumulative effects of DON on fish health and performance demonstrate the need for effective mitigation strategies in aquaculture. Mitigation approaches, such as yeast cell wall extracts and commercial feed additives show promise in reducing the adverse effects of DON contamination (Hooft and Bureau 2017; Wang et al. 2021). However, further research is needed to expand our understanding of DON’s full range of effects across different fish species and to develop targeted, evidence-based mitigation strategies.

### Ochratoxin A

Ochratoxin A (OTA), a secondary metabolite produced by various species of fungi, is well recognized as an undesired contaminant in both domesticated and farmed-animal feed, and it also poses significant threats to aquatic organisms. Harmful effects have been documented in many fish species commonly reared in aquaculture (Table 7), including rainbow trout (*Oncorhynchus mykiss*), European seabass (*Dicentrarchus labrax*), tambaqui (*Colossoma macropomum*), Atlantic salmon (*Salmo salar*), grass carp (*Ctenopharyngodon Idella*), Nile tilapia (*Oreochromis niloticus*), channel catfish (*Ictalurus Punctatus*), and Eurasian carp (*Cyprinus carpio*).

**Table 7:** Effects of ochratoxin A on aquaculture fish species

Fish species	Dose	Exposure period	Physiological effect	Biochemical effect	Reference
*Oncorhynchus mykiss*	2–8 mg/kg body weight	10 days (intraperitoneal injection)	increased mortality, oedema and hemorrhages in the visceral fat; swollen and discolored liver; pale kidney	NM	Doster et al. 1972
*Dicentrarchus labrax*	50–400 µg/kg body weight	96 h (oral exposure)	altered behavior (sluggish movement, loss of equilibrium), rapid operculum movement, and appearance of hemorrhagic patches on the dorsal surface	NM	El-Sayed et al. 2009
*Colossoma macropomum*	1.6–2.4 mg/kg feed	14 days (oral exposure)	decreased weight, WG, and liver weight	disrupted energy metabolism pathways in the liver	Baldissera et al. 2020
*Salmo salar*	0.2–2.4 mg/kg feed	8 weeks (oral exposure)	statistically unclear effect of OTA exposure on growth performance indicators	increased expression of IFNγ at 3 weeks; increased expression of Ki67 at 6 weeks indicating proliferation of immune cells in the spleen; reduced blood levels of TGs, TP and albumin	Bernhoft et al. 2018
*Ctenopharyngodon idella*	406–2406 µg/kg feed	60 days (oral exposure)	retarded growth and disrupted intestinal structural integrity; decreased feed efficiency, percentage WG and SGR	oxidative damage and increased intestinal permeability; induced RhoA/ROCK signaling pathway; damaged intestinal apical junctional complexes	Liu et al. 2021
*Oreochromis niloticus*	0.5 mg/kg feed	10 weeks (oral exposure)	sluggish swimming; reduced survival and growth performance; post-mortem lesions in liver, kidneys and spleen	increased serum levels of ALT, AST, creatinine and urea; reduced serum levels of TP, albumin and globulin	Diab et al. 2018
*Ictalurus punctatus*	2.0–4.0 mg/kg feed	6 weeks (oral exposure)	decreased weight; increased mortality after challenge with E*. ictaluri*	NM	Manning et al. 2005
*Ictalurus punctatus*	0.5–8.0 mg/kg feed	8 weeks (oral exposure)	increased mortality; reduced WG and feeding efficiency, lowered Ht; melanomacrophage centers occurred in hepatopancreatic tissue and posterior kidney	NM	Manning et al. 2003
*Cyprinus carpio*	1.85 mg/kg body weight	24 h (oral exposure)	NM	increased conjugated dienes and ROS; decreased expression of genes regulating oxidative stress (*keap1, gpx4b*, *gpx4b*)	Kövesi et al. 2020
*Incalurus punctuatus*	2–8 mg/kg feed	56 days (oral exposure)	decreased FI and increased feed conversion ratio; increased susceptibility to *Saprolegnia* challenge	statistically unclear effect of exposure on AMPP expression	Zahran et al. 2016
*Danio rerio*	0.1–0.5 µM	8 days (waterborne exposure)	increased mortality of embryos; anatomopathological changes (pericardial edema and dark yolk sac); reduced size of developing liver	decreased expression of genes encoding coagulation factors and liver markers (*f7, f9b, cp, vtna*)	Wu et al. 2018
*Danio rerio*	1.38–5.53 mg/kg body weight	96 h (intraperitoneal injection)	altered swimming behavior	increased levels of oxidative stress-related proteins (GPx, GST, GR) and decreased NPSH levels in the brain	Valadas et al. 2023
*Danio rerio*	0.25–0.5 µM	72 h (waterborne exposure)	intracerebral hemorrhage; disrupted vascular patterning; cavity-like pattern in the hindbrain ventricles, implying the possibility of cerebral edema	increased expression of *miR-731*	Wu et al. 2019
*Danio rerio*	0.05–2.5 μM	5 days (waterborne exposure)	embryo deformities; reduced growth and hatching rates; increased mortality	NM	Haq et al. 2016
*Ctenopharyngodon idella*	406–2406 μg/kg feed	60 days (oral exposure)	OTA residues in muscle; altered musculature (decreased muscle fiber diameter and density, muscle fiber breakage); muscle oxidative damage and endoplasmic reticulum stress; reduced muscle protein and collagen deposition	oxidative stress reduced the activity of antioxidant genes (*GPx1*, *Trx*) by inhibiting the *PGC1-α/Nrf2* pathway; increased expression of stress response genes (*GRP78, eIF2α, ATF6, PERK, CHOP*); blocked protein synthesis and promoted expression of genes related to protein breakdown; reduced collagen production by lowering the activity of genes involved in tissue repair and fibrosis control (*TGF-β1, Smad, CTGF, LOX*)	Zhao et al. 2023
*Ctenopharyngodon idella*	406–2406 μg/kg feed	60 days (oral exposure)	histopathological changes in the spleen and head kidney (decreased number of lymphocytes and necrotizing renal parenchymal cells)	oxidative damage reduced the activity of antioxidant enzymes (*GPX1*, *GPX4*, *GSTO*) by suppressing the Nrf2 pathway; apoptosis triggered by the mitochondria and death receptor pathways; reduced expression of anti-inflammatory genes (*IL-10*, *IL-4*) and increased expression of pro-inflammatory genes (*TNF-α*, *IL-6*), indicating a shift toward inflammation and oxidative stress	Zhao et al. 2022a, b

Negative effects of OTA exposure have been demonstrated in different fish but these studies tend to focus on different outcomes. Under oral exposure to OTA-contaminated feed, fish mostly display decreased weight gain and thus lower final mean weight. These effects have been documented in channel catfish, tambaqui, and grass carp (Baldissera et al. 2020; Liu et al. 2021; Manning et al. 2005).

However, the exposure to OTA can also affect the behavior of fish. In sea bass reared in marine water (*Dicentrarchus labrax*), OTA exposure led to sluggish movement, loss of equilibrium, rapid operculum movement, and hemorrhagic patches on the dorsal surface (El-Sayed et al. 2009). These alterations are primarily ascribed to the potent acute neurotoxic and damaging oxidative effects of OTA (Sava et al. 2006). Moreover, alterations in antimicrobial polypeptide expression and resistance to water mold infection have been reported in channel catfish (*Ictalurus punctatus*) (Zahran et al. 2016), implicating the ability of OTA to affect the immune system.

At the cellular and molecular levels, OTA triggers apoptosis in muscle tissue through both mitochondria and death receptor pathways, potentially via activation of p38 mitogen-activated protein kinase (*p38MAPK*), as demonstrated in grass carp. Furthermore, OTA exacerbates inflammation by downregulating anti-inflammatory cytokines (e.g., IL-10, IL-4) and upregulating pro-inflammatory factors (e.g., TNF-α, IL-6), which could be mediated by the Janus kinase/signal transducer and activator of the transcription (JAK/STAT) signaling pathway (Zhao et al. 2022a). These effects demonstrate the pro-inflammatory properties of OTA leading to developmental disorders of juvenile fish, and probably affecting the fish behavior.

The effects of OTA exposure can also manifest as histological alterations in fish tissues. In Atlantic salmon (*Salmo salar*), OTA induces histopathological changes in the liver and intestine associated with reduced TGs, total protein (TP), and albumin (Bernhoft et al. 2018). Similarly, in Nile tilapia (*Oreochromis niloticus*) histological lesions in the liver, kidney, and spleen occur following experimental ochratoxicosis (Diab et al. 2018). Histopathological examination of OTA-exposed grass carp also decreases muscle fiber diameter and density, considered to be effects of oxidative stress and reduced muscle protein and collagen deposition. At the molecular level, the oxidative stress is be attributed (at least in part) to downregulated expression of antioxidant enzymes like *GPx1* and *Trx*. Additionally, OTA exposure induces endoplasmic reticulum expansion and upregulated expression of stress markers, including *GRP78, eIF2α, ATF6, PERK,* and *CHOP* (Liu et al. 2021; Zhao et al. 2022b).

Importantly, much of the information on the biological properties of OTA originates from experiments conducted not on commonly farmed aquaculture species, but rather on a well-examined laboratory fish model. As such, in zebrafish (*Danio rerio*) embryos, OTA triggers disruption in liver development and interferes with the formation of coagulation factors (Wu et al. 2018). Moreover, the fish embryos exposed to OTA exhibit discernible deformities, reduced growth and hatching rates, and increased lethality (Haq et al. 2016). Research on the molecular background of OTA toxicity shows that the mycotoxin is able to modulate microRNA levels and affect prolactin receptor signaling pathways implicated in intracerebral hemorrhage in zebrafish embryos (Wu et al. 2019). Increased levels of glutathione peroxidase (GPx), glutathione-S-transferase (GST), and glutathione reductase (GR), along with decreased non-protein thiol (NPSH) levels in the brain correlate with the zebrafish behavior altered by the exposure to OTA (Valadas et al. 2022).

In summary, the available research demonstrate that feed contamination with OTA poses a threat to fish welfare. Across different species that are popular in aquaculture, the exposure to OTA leads to reduced growth, altered behavior, and compromised immune function. At the cellular and molecular levels, the mycotoxin is able to trigger apoptosis, evoke inflammation and oxidative stress, causing histopathological changes in organs such as the liver, kidney, spleen, or muscle. Together with research on OTA-induced malformations in developing zebrafish embryos, these studies call for effective feed monitoring and regulatory measures to ensure the welfare of fish populations reared under aquaculture conditions.

### Fumonisin B1

Among the different FUM species, fumonisin B1 (FB_1_) in particular has gained considerable attention due to its adverse effects on animal health. The presence of FB_1_ in fish feed should also be of concern for the aquaculture industry, as fish, like other vertebrates, are also susceptible to this mycotoxin.

With a few exceptions, the studies in the literature have focused on the biological effects of FB_1_ rather than those of other FUM derivatives, and most of those studies have reported that FB_1_ impairs fish growth (Table 8). For example, the growth and feed efficiency of channel catfish (*Ictalurus punctatus)* fed an FB_1_-contaminated diet is significantly reduced (Lumlertdacha et al. 1995). Similarly, Nile tilapia (*Oreochromis niloticus*) display dose-dependent reductions in growth performance, reflecting the negative influence of FB_1_ on fish growth and pisciculture productivity (Tuan et al. 2003). Additionally, Olatunde Oginni et al. (2020) investigated the influence of FB_1_-contaminated diets on the slaughter value and proximate composition of *Clarias gariepinus* flesh, revealing potential implications in terms of fish quality and decreased nutritional value as the FB_1_ concentration increases.

**Table 8:** Effects of fumonisin B1 on aquaculture fish species

Fish species	Dose	Exposure period	Physiological effect	Molecular process	Reference
*Ictalurus punctatus*	0.3–720 mg/kg feed	10 and 14 weeks (oral exposure)	reduced WG; hematological alterations (decreased Ht and RBCs count, altered white blood cell counts); reduced feed consumption; swollen hepatocytes with lipid-containing vacuoles; lymphocyte infiltration; scattered necrotic hepatocytes	NM	Lumlertdacha et al. 1995
*Oreochromis niloticus*	10–150 mg/kg feed	8 weeks (oral exposure)	reduced WG and Ht; increased free spinganine to free sphingosine ratio in the liver	NM	Tuan et al. 2003
*Oreochromis niloticus*	50 mg/kg feed	20 days (oral exposure)	reduced WG and FC	reduced mRNA expression of GHR and IGF-1 engaged in promotion of growth	Claudino-Silva et al. 2019
*Rhamdia quelen*	6.2 mg/kg feed	30 days (oral exposure)	reduced weight, GR and WG; increased FCR	hallmarks of oxidative stress in the brain (increased ROS, LOOH and protein carbonylation in the brain) while decreased activity of antioxidant response elements (ACAP, CAT, GPx, GST)	Baldissera et al. 2020
*Clarias gariepinus*	2.5–7.5 mg/kg feed	12 weeks (oral exposure)	decreasing slaughter value with increasing FB_1_ dose	NM	Oginni et al. 2020
*Cyprinus carpio*	10–100 mg/kg feed	42 days (oral exposure)	vacuolated, degenerated, or necrotic neural cells scattered around damaged blood capillaries and in the periventricular area in the brain	NM	Kovačić et al. 2009
*Oreochromis niloticus*	20–60 mg/kg feed	30 days (oral exposure)	reduced WG, FE	reduced mRNA expression of GHR and IGF-1 engaged in promotion of growth	Claudino-Silva et al. 2018
*Cyprinus carpio*	0.5–5.0 mg/kg feed	42 days (oral exposure)	loss of body weight; higher incidence of infective bacterial dermatological lesions (erythrodermatitis cyprinid); increased RBCs and platelet counts; increased activity of AST and ALT; increased levels of creatinine and bilirubin in serum	NM	Pepeljnjak et al. 2003
*Cyprinus carpio*	10–100 mg/kg feed	42 days (oral exposure)	reduced WG; dermal lesions (erythrodermatitis cyprini); edematous and pale liver with enlarged gall blader and dark and viscous bile; pale and edematous head and trunk kidney; edematous and hyperemic bulbus arteriosus	NM	Petrinec et al. 2004
*Ictalurus punctatus*	1–10 mg/kg body weight/day	21 days (intraperitoneal injection)	dose-dependent intracellular organelle alterations (nucleus, nucleolus, granular endoplasmic reticulum, and mitochondria); alterations and disruption of cellular membranes; necrosis and apoptosis in liver	NM	Scaff And Scussel 2008
*Cyprinus carpio*	1.96 mg/kg body weight	single oral dose (oral exposure)	decreased GSH concentration in liver	induced oxidative stress (increased MDA content), while decreased GSH content and GPx activity in the liver	Kövesi et al. 2020

The effect of FB_1_ on fish growth is likely linked to disruption of growth regulatory pathways. For example, Nile tilapia fingerlings challenged with FB_1_ exhibited changes in expression of insulin-like growth factor 1 (IGF-1) and growth hormone receptor (GHR) mRNA in their liver, pointing to the molecular mechanism responsible for growth hormone pathway disruption (Claudino-Silva et al. 2018).

However, hematological and histopathological alterations have also been found in FB_1_-exposed channel catfish, indicating that this mycotoxin is capable of damaging and inducing dysfunction in fish livers (Cirra Scaff and Scussel 2008). Moreover, FB_1_ exhibits neurotoxic effects in fish, such as young carp (*Cyprinus carpio*). For example, chronic exposure of these fish to FB_1_ resulted in vacuolated, degenerate, or necrotic neural cells in the brains, indicating that the toxin permeated the blood–brain barrier and caused significant neuronal damage (Kovačić et al. 2009). It is believed that these effects may be due to induction of oxidative stress (Baldissera et al. 2020).

### Toxin T-2

Toxin T-2, a potent secondary metabolite produced by various *Fusarium* species, has garnered attention due to its adverse effects on aquatic organisms, particularly fish. Studies has shown various detrimental impacts of T-2 toxin on fish health (Table 9), ranging from oxidative stress and developmental abnormalities to increased mortality rates in common carp (*Cyprinus carpio*), rainbow trout (*Oncorhynchus mykiss*), channel catfish (*Ictalurus punctatus*), and zebrafish (*Danio rerio*).

**Table 9:** Effects of toxin T-2 on aquaculture fish species

Fish species	Dose	Exposure period	Physiological effect	Biochemical effect	Reference
*Cyprinus carpio*	5.3 mg/kg feed	4 weeks (oral exposure)	hematological alterations (decreased Ht and RBCs count, leading to anemia, and reduced white blood cell counts, leading to leukopenia)	changes in non-specific immune response and cytokine levels in the head kidney (increased expression of TNF-a and IL-10 in the head kidney); hallmarks of oxidative stress (increased lipid peroxidation) in the liver and caudal kidney; changed activity of antioxidant response elements (GST, CAT, GPx) in the liver and caudal kidney; increased ceruloplasmin activity in blood	Matejova et al. 2017
*Oncorhynchus mykiss*	1.0–1.8 mg/kg feed	4 weeks (oral exposure)	decreased TP, ALB, and ammonium in plasma	induced oxidative stress (increased lipid peroxidation) and antioxidant defenses in the liver (increased activity of GST, GSR, and GPx, decreased activity of CAT)	Modra et al. 2018
*Ictalurus punctatus*	1.0–2.0 mg/kg feed	6 weeks (oral exposure)	reduced WG; increased mortality after *E. ictaluri* challenge	NM	Manning et al. 2005
*Ictalurus punctatus*	0.625–5.0 mg/kg feed	8 weeks (oral exposure)	reduced growth; reduced feed conversion; decreased Ht, gastric lesions; decreased cellularity of the hematopoietic areas in the head kidney	NM	Manning et al. 2003
*Danio rerio*	0.05–0.80 µmol/L	short exposure at early embryonic developmental stage (4–6 hpf) (waterborne exposure)	excessive apoptosis; increased mortality; developmental malformations (abnormalities in tail formation, cardiovascular defects) and behavioral changes (side-wise position and lack of swimming behavior)	increased levels of ROS	Yuan et al. 2014
*Oncorhynchus mykiss*	1.0–15 mg/kg feed	16 weeks (oral exposure)	decreased growth, feeding efficiency, Ht, blood Hb concentration, and feed acceptance; transitory edema	NM	Poston et al. 1982
*Oncorhynchus mykiss*	200–400 ppm	12 months (oral exposure)	pronounced tissue irritation; exposure to a low dose had a growth-promoting effect	NM	Marasas et al..^1969^

Foundational studies by Marasas et al. (1969) and Poston et al. (1982) provided early insights into the biological effects of dietary T-2 toxin on rainbow trout. Those authors observed histopathological alterations in organs, hematological abnormalities, and systemic toxicity, pointing to the wide range of deleterious effects of T-2 toxin on fish health. Further studies have shown that dietary exposure to T-2 toxin increases fish mortality rates. This effect has been observed in T-2 toxin-exposed channel catfish, particularly when challenged with bacterial pathogens such as *Edwardsiella ictaluri* (Manning et al. 2003, 2005).

The potential of a synergistic interaction between T-2 toxin and pathogenic microorganisms has led to follow-up studies aiming to unravel the mechanisms of the observed immunosuppression. In common carp and rainbow trout, oxidative stress induced by T-2 toxin disrupts antioxidant defense mechanisms, leading to accumulation of ROS, which causes oxidative damage to vital biomolecules, like DNA or cellular membranes. This has been implicated in impairing immune function, compromising cellular integrity, and affecting the overall health and survival of fish (Matejova et al. 2017; Modra et al. 2018).

Oxidative stress is also believed to be the main molecular mechanism responsible for the teratogenic properties of T-2 toxin in fish. For example, in zebrafish embryos, T-2 toxin induces apoptosis and developmental defects (cardiovascular defects, abnormalities in tail formation), indicating the vulnerability of early life stages to T-2 toxin (Yuan et al. 2014).

### Emerging mycotoxins

Beauvericin (BEA) is among the most studied emerging mycotoxins and has been shown to negatively affect fish growth. In experiments with fish, exposure to this mycotoxin decreased growth, protein digestion, and feed conversion rate (Berntssen et al. 2023). This indicates that it can significantly impair fish metabolism, limiting their ability to utilize nutrients efficiently. Enniatin B (ENB), another emerging mycotoxin, also impaired growth, though this effect was not related to feed conversion efficiency. Similarly, moniliformin reduced weight gain in Nile tilapia and channel catfish (Yildirim et al. 2000; Tuan et al. 2003).

The effects of these toxins extend beyond growth and can also include significant physiological damage. ENB induces anemia in fish, while BEA triggers a pronounced oxidative stress response (Berntssen et al. 2023). This suggests that these toxins can have widespread effects on fish health by disturbing critical physiological processes such as oxygen transport and cellular defense mechanisms. In another example, exposure of zebrafish embryos to alternariol (AOH) increased mortality and caused severe developmental defects in survivors, including tail malformations, edema, uninflated swim bladders, and body axis curvature (Fliszár-Nyúl et al. 2022).

BEA and ENB both induced significant disturbances in energy metabolism and oxidative stress in primary hepatocytes isolated from Atlantic salmon, leading to imbalances in iron homeostasis and increased sensitivity to ferroptosis (Søderstrøm et al. 2022). These findings show that mycotoxins can cause damage at the cellular level, affecting both organ function and cellular survival mechanisms.

Studies in other vertebrate models also highlight the potential risks of emerging mycotoxins. For example, rodent studies suggest that alternariol monomethyl ether (AME) can cause liver, kidney, and spleen damage, with additional genotoxic effects such as gene mutations, chromosome breakage, and DNA damage (Tang et al. 2022). Given these results, it is critical to conduct more species-specific toxicity assessments in fish to better understand the interspecies variability in response to mycotoxin exposure. Such research could lead to the development of more tailored strategies for mitigating the harmful effects of these compounds in different fish species.

The varying effects of emerging mycotoxins across different fish species likely reflect the diversity in piscine metabolism, physiology, and aquaculture husbandry requirements. While some species may exhibit heightened sensitivity to certain toxins, others might naturally display more resilience due to their feeding preferences towards plant ingredients. Therefore, continued research on species-specific toxicity profiles and the underlying molecular mechanisms is essential for safeguarding aquatic health and developing effective mitigation strategies.

Despite growing awareness of the presence of emerging mycotoxins in aquatic environments and their potential effects on fish, several knowledge gaps persist. Limited information is available on the uptake, distribution, metabolism, and elimination of emerging mycotoxins in fish. Understanding the toxicokinetics of all these compounds is essential for assessing their bioavailability and the risks resulting from their potential for bioaccumulation in the edible fish tissues. While acute toxicity studies have provided valuable insights into the adverse effects of emerging mycotoxins on fish, more research is needed to elucidate sublethal effects, including reproductive impairment, immunotoxicity, and neurotoxicity. Long-term, chronic exposure studies could also help assess chronic effects and cumulative toxicity over time.

## Insects as potential intermediate vectors of feed contamination with mycotoxins

Fish were the first group of animals for which the European Union permitted the inclusion of insect meal and oil as feed ingredients. This change in legislation came into effect in July 2017, allowing the use of processed animal proteins (PAPs) from insects in aquaculture. This decision was driven by the need to reduce reliance on traditional fishmeal and fish oil in aquaculture feed, which was becoming increasingly unsustainable due to the overfishing of natural populations and other environmental concerns. Insects such as black soldier fly larvae, mealworms, and house crickets are now authorized as part of this alternative feed source (Commission Regulation (EU) 2017/893) due to their high nutritional value and because they can efficiently convert organic material into valuable protein (Barragan-Fonseca et al. 2017; Zielińska et al. 2015).

The growing interest in utilizing insects as a sustainable protein source in animal feed (Hong et al. 2020), particularly in aquaculture, has prompted the investigations of their role as intermediaries in the transfer of harmful substances, including mycotoxins from plant-based insect-feeding material to animal feed. However, detailed studies on the toxicokinetics of mycotoxins in insects remain limited to only a few compounds and the most popular insect species. Moreover, most studies describing mycotoxin transfer from feed into insect bodies and frass report large discrepancies in the mycotoxin mass balance, i.e. the difference between the mycotoxin content in the feed and the total sum of the parent compounds and their known metabolites in the insect bodies and frass (Niermans 2024). Such incomplete understanding of mycotoxin metabolism in insects reared for feed poses a potential risk to the welfare of animals fed mycotoxin-contaminated insect meal or oil.

### Biotransformation of Mycotoxins in Insects

The toxicokinetics of mycotoxins in insects involves the processes of absorption, distribution, metabolism, and excretion, which determine the fate and biological effects of these harmful substances in insect bodies. Understanding these processes is crucial for the assessment of possible risks, as they not only influence insect physiology but may also have implications for mycotoxin transfer to edible fish products (Kępińska-Pacelik and Biel 2022).

Upon ingestion of contaminated plant material, mycotoxins can be absorbed through the insect gut lining. The rate and extent of absorption vary depending on factors such as the physicochemical properties of the mycotoxin, the insect species, or the composition of the feed substrate (Bisconsin-Junior et al., 2023; Niermans et al. 2021a). Once absorbed, mycotoxins may undergo biotransformation within the insect’s body. These metabolic processes often lead to the formation of metabolites that are easier to transport or elimination (Birnbaum and Abbot, 2018). In many cases, these insect-derived mycotoxin metabolites exhibit reduced toxicity compared to their parent compounds (Niermans et al. 2021b; Bisconsin-Junior et al., 2023).

It is generally postulated that most insects do not accumulate significant amounts of mycotoxins, as they have efficient metabolic and excretory mechanisms to eliminate these compounds to their feces (Niermans et al. 2021a, b; Camenzuli et al. 2018; Schrögel and Wätjen 2019). However, although some studies have already explored the toxicokinetics of mycotoxins of most concern such as DON, OTA or FB_1_ in insects, detailed toxicokinetic experiments demonstrating a complete mass balance of the mycotoxin used in the study are currently available only for AFB_1_ (Niermans et al. 2024).

The insect metabolism of AFB_1_ is among the best studied (Niermans et al. 2024). Insects metabolize AFB_1_ into hydroxylated metabolites via NADPH-dependent reductases and hydroxylases, generally producing metabolites with reduced toxicity compared to the parent compound (Camenzuli et al. 2018; Evans and Shao 2022). For example, yellow mealworm (YMW; *Tenebrio molitor*) has been shown to convert AFB_1_ into aflatoxin M1 (AFM_1_), a hydroxylated metabolite (Bosch et al. 2017). Similarly, the same metabolite has been detected in the frass of another insect – lesser mealworm (LMW; *Alphitobius diaperinus*) fed with AFB1-contaminated substrate (Camenzuli et al. 2018). In contrast, black soldier fly (BSF; *Hermetia illuscens*) larvae were able to produce another metabolite, namely aflatoxin P1 (AFP_1_), which was found in their residual material (spiked feed/frass). In turn, both LMW and BSF yielded aflatoxicol (a reduced metabolite) in the residual material, which was observed at higher exposure levels (Camenzuli et al. 2018). Importantly, these insect-derived metabolites have been usually found in trace contents and they are generally considered to be less toxic than AFB_1_, i.e. exhibiting lower acute toxicity and genotoxicity than the parent toxin (Eaton et al. 2025). Finaly, in a study on navel orangeworm (*Amyelois transitella*) and codling moth (*Cydia pomonella*) exposed to AFB_1_, larvae of neither of the two insect species were able to produce AFB_1_−8,9-epoxide, a highly-reactive metabolite (Lee and Campbell 2000), which is in fact, responsible for the aflatoxin carcinogenicity (Guengerich et al. 1998). These results altogether confirm that the insect metabolism of AFB_1_ is oriented towards its excretion or degradation rather than bioactivation, providing a promising outlook for the safety of insects used as fish feed ingredients (Niermans et al. 2024).

In addition to AFB_1_, metabolism of ZEN in insects has also been described, but to a lesser extent. Studies on YML and/or BSF show that ZEN largely does not accumulate in larval bodies (Leni et al. 2021; Niermans et al. 2019). However, ZEN derivatives, α-zearalenol (α-ZEL) and β-zearalenol (β-ZEL), have been detected in BSF larvae (Camenzuli et al. 2018), and distinguishable levels of these metabolites have been found in YMW frass fed on ZEN-spiked substrate (Niermans et al. 2019), indicating an active metabolism of this mycotoxin in insects. Among these two compounds, α-ZEL exhibits a higher affinity for estrogen receptors than ZEN or β-ZEL, making it a more potent estrogen agonist (e.g. Tatay et al. 2018). The increased estrogenic activity of α-ZEL raises concerns about the potential to disrupt endocrine systems in animals that consume insects containing these metabolites (Evans and Shao 2022; Camenzuli et al. 2018). However, because these metabolites are mainly not retained in the insect larvae, the potential risk would rather come from exposure to the frass or to larvae containing unemptied gut contents. To minimize this risk, good hygiene practices on insect rearing already include a fasting period to clear larvae’s gut before their harvest (IPIFF, 2024).

In the case of DON, insects do not show high accumulation of this mycotoxin but rather high tendency to excrete it in their frass. For example, in a feeding trial on YML larvae exposed to DON-spiked diet (groups with increasing content from 2 to 12 mg/kg), the larvae had the same DON content (from 0.122 to 0.136 mg/kg) across all diet groups, unlike the its content in frass which was increasing with the increase of DON the insects’ diet (Sanabria et al. 2019a). This suggests that DON may not accumulate proportionally with the dietary levels (Sanabria et al. 2019b). Studies on BSF also showed only minimal (if any) absorption of DON in the larval bodies (Camenzuli et al. 2018; Leni et al. 2019). Insects convert DON into less toxic derivatives, including 3-acetyl-DON, 15-acetyl-DON and DON-3-glycoside, which are also more readily excreted than the parent compound (Evans and Shao 2022; Camenzuli et al. 2018; De Zutter et al. 2016).

Insects possess gut microbiota capable of degrading toxic compounds. For instance, the yeast *Apiotrichum mycotoxinivorans*, isolated from the gut of the termite *Mastotermes darwiniensis*, can degrade OTA into metabolites exhibiting reduced toxicity in vitro (Molnar et al. 2004) One of the documented pathways of OTA detoxification involves the hydrolysis of the ester bond, catalyzed by esterases, leading to the formation of ochratoxin α (OTα), which possesses significantly lower toxicity. The enzymes implicated in this transformation include carboxypeptidase A, carboxypeptidase PJ_1540, protease A, lipase A, and ochratoxinase (Dobritzsch et al. 2014; Liuzzi et al. 2017; Stander et al. 2000).

The detoxification of other mycotoxins, such as FUMs, involves the activity of enzymatic systems, including cytochrome P450 monooxygenases (CYPs) and glutathione S-transferases (GSTs) (Zhang et al. 2017). CYPs catalyze oxidative metabolism reactions of xenobiotics by introducing polar functional groups, consequently increasing their solubility and facilitating excretion. Concurrently, GSTs participate in the detoxification of FUMs by conjugating glutathione to reactive intermediate metabolites, enabling their effective removal from the insect body (Heidel-Fischer and Vogel 2015; G. Zhang et al. 2024a, b).

Despite the limited direct evidence for the metabolism of OTA and FUMs by enzymes that are directly produced by insects, the symbiotic microorganisms residing in their gut appear to play a substantial role in these processes. This could explain why bioaccumulation risk assessment demonstrated that *T. molitor* larvae did not accumulate OTA or FB_1_ in detectable or dangerous concentrations (Mancini et al. 2020).

As mentioned above, a major challenge in the study of mycotoxin toxicokinetics in insects is the mass balance discrepancy observed in various experiments. Majority of these studies indicate that only a small fraction of the initial mycotoxin load remains detectable in residual feed and frass (Bosch et al. 2017; Purschke et al. 2017; Camenzuli et al. 2018; Niermans et al. 2019). Consistently, a systematic review of the literature demonstrates that a large portion of the mycotoxin mass ingested by insect larvae is usually unaccounted for (Niermans et al. 2021a, b). For example, in BSF fed with DON-spiked diets, the quantified parent compound and its known metabolites accounted for only part (from 39 to 55%) of the mycotoxin mass balance (Camenzuli et al. 2018); and in the case of AFB1-exposed BSF larvae, the recovery of the parent compound and its metabolites ranged between 11 to 18% (Camenzuli et al. 2018). Likewise, the overall mycotoxin mass balance in LMW fed with naturally-contaminated substrate with DON, FUMs and ZEN did not exceed 60%, demonstrating that mycotoxins must be metabolized by insects at least partially to unknown compounds (Leni et al. 2019). On the other hand, in the recent study on BSF larvae fed with naturally-contaminated peanut press cake, a full recovery of the initial AFB1 mass was demonstrated, mostly in the insect frass (Niermans et al. 2024).

While some authors propose that the unaccounted fraction of mycotoxins from the initial mass may be degraded or transformed within the insect gut into unidentified metabolites or lost due to matrix interference with the feed substrate or insect cuticle, the ultimate fate of these compounds remains unclear (Camenzuli et al. 2018; Leni et al. 2019). The prevailing hypothesis emphasizes the role of gut microbiome in the mycotoxin degradation, with its composition largely influenced by the insect’s diet (Niermans et al. 2024). However, more precise investigation is needed to clarify the metabolic pathways involved and to determine whether these undetected metabolites (if exist) are fully excreted or retained within insect tissues. Importantly, toxicological studies evaluating the safety of using mycotoxin-exposed larvae as components of feed have yet to provide any evidence on the potential effects of these metabolites in animals (Bittner, in preparation). In the case of lack of consensus on the metabolic gap, such studies could be essential to assess the potential risks associated with insect-based feed derived from contaminated substrates.

To summarize, mycotoxins generally exert minimal effects on insect growth and survival, and the available literature on this topic indicates that their accumulation in commonly farmed insect species for feed components (like BSF or YMW) is low. While insects possess metabolic pathways that allow for mycotoxin biotransformation, the extent of this metabolism varies depending on the specific mycotoxin, feeding substrates and insect species. Many studies highlight the potential of insects as an alternative source of fat and protein of good quality. However, due to the incomplete understanding of insect metabolic pathways, insect feed substrates of poor quality should be avoided. This concern is particularly emphasized by the “metabolic gap” observed in multiple studies, where in many studies substantial portions of ingested mycotoxin mass remain unaccounted for. Furthermore, some mycotoxin metabolites retain biological activity and may have toxic effects. Thus in practice, a fasting period for insects before harvest is practiced to clear gut contents to mitigate potential risks. To fully understand the threats resulting from the insect-feed contamination with mycotoxins, future studies should focus on the identification, quantification, and toxicity assessment of these undetermined insect metabolites of mycotoxins.

## Conclusions

Although it is impossible to guarantee that none of the still-unknown mycotoxin metabolites will ever prove harmful, the weight of current evidence provides only limited cause for concern regarding finished fish products. However, it must be emphasized that the available data represent only a fraction of global aquaculture production — both in terms of species diversity and geographical coverage. The vast majority of farmed fish are produced in tropical and subtropical regions, where feed materials of local origin are often poorly surveyed for mycotoxin contamination and environmental conditions favor fungal growth. Studies conducted over the past decade have consistently shown three things: (a) raw plant-derived ingredients, especially cereals, oilseeds and certain legumes, carry mycotoxins at concentrations high enough to impair growth, immunity and reproduction in several farmed fish species; (b) once these ingredients are processed into commercial diets (whether conventional or enriched with alternative protein and fat sources, such as insect meal and oil) the detected levels fall well below the EU guidance values; and (c) mycotoxin residues in edible fillets are generally at, or below, the limits of analytical detection. In other words, the aquaculture production chain already reduces contamination step-by-step: there is a relatively large amount in raw materials, far less in feed, and negligible amounts in fish on the plate.

Nevertheless, it should be noted that current EU and FDA guidance values are based on limited data and address only a subset of regulated mycotoxins. Co-occurrence and potential synergistic effects among toxins, as well as interspecific differences in sensitivity, remain largely unexplored, and may render these thresholds conservative for some aquaculture species. Furthermore, while contamination in edible fillets appears negligible, certain species are consumed whole (including viscera and liver) which may alter real exposure levels in human consumers.

However, this reassuring picture is shadowed by a mass-balance puzzle: the quantities of toxin that disappear between the grain silo and the market stall are only partly explained by known mechanisms of degradation, binding, or excretion. Untangling the fate of this missing fraction is now the most pressing scientific question, and the answer will dictate how confidently novel plant- and insect-based feed ingredients can be scaled up.

The most obvious knowledge gaps involve metabolism. We still lack a full map of how common aquaculture species absorb, transform, and eliminate parent mycotoxins and their masked, plant-modified metabolites with unknown toxicological profiles. Likewise, the biotransformation pathways that operate when black soldier fly larvae, mealworms, or other insects ingest contaminated substrates remain largely uncharted. Filling these gaps will require (i) analytical methods that can detect an expanded suite of known and hypothesized metabolites at trace levels, and (ii) long-terms studies that track their kinetics and biological effects under realistic fish-farming conditions.

From a practical standpoint, three actions immediately follow from this. First, routine surveillance must stay focused on raw ingredients, because that is where the risk is greatest, and where mitigation, via sourcing, storage, and blending, offers the greatest benefits. Second, feed formulators should continue exploiting the natural “dilution–detoxification” effect of processing while validating that new alternative ingredients do not reintroduce unacceptable loads of toxins. Third, researchers, regulators, and industry need to collaborate on open-access databases that couple occurrence data with toxicokinetic and toxicodynamic endpoints, so that emerging hazards can be predicted rather than merely detected.

In summary, while the general pattern of decreasing mycotoxin levels at each step of the aquaculture feed chain is reassuring, the true extent of risk remains incompletely characterized. Expanding our knowledge beyond the currently studied species and production systems will be essential for refining regulations, developing reliable monitoring frameworks, and ensuring feed and food safety under increasingly variable environmental conditions.

## Data Availability

No datasets were generated or analysed during the current study.

## Abbreviations

A:G: Albumin:globulin ratio
AChE: Acetylcholinesterase
ACP: Acid phosphatase
AFB_1_: Aflatoxin B1
AFL: Aflatoxicol
AFM_1_: Aflatoxin M1
AFQ_1_: Aflatoxin Q1
AFs: Aflatoxins
ALP: Alkaline phosphatase
ALT: Alanine aminotransferase
AME: Alternariol monomethyl ether
Ampk: SIRT1/5′-AMP-activated protein kinase
AMPs: Antimicrobial peptides
AOH: Alternariol
Apaf-1: Apoptotic protease activating factor-1
ASI: Adipose somatic index
AST: Aspartate aminotransferase
Bax: Bcl2-associated X protein
BEA: Beauvericin
C3: Complement 3 protein
C4: Complement 4 protein
CAT: Catalase
CP: Crude protein
CYP450: Cytochrome P450
DON: Deoxynivalenol
DON-3Glc: Deoxynivalenol-3-glucoside
ENA_1_: Enniatin A1
ENB_1_: Enniatin B1
ERE: Energy retention efficiency
EROD: 7-Ethoxy-resorufin-O-deethylase
ERs: Estrogen receptors
FasL: Fas ligand
FB_1_: Fumonisin B1
FB_2_: Fumonisin B2
FBW: Final body weight
FCR: Feed conversion ratio
FI: Feed intake
FUMs: Fumonisins
GSH: Glutathione
GSH-Px: Glutathione peroxidase
GSI: Gonadosomatic index
GST: Glutathione S-transferase
Hb: Hemoglobin
Hpf: Hours post fertilization
HSI: Hepatosomatic index
Ht: Hematocrit
HT2-3Glc: HT-2-toxin-3-glucoside
IFN1: Type-I interferon
IFNγ: Interferon γ
IgM: Immunoglobulin M
IL: Interleukin
IL-1β: Interleukin 1β
inf-α: Interferon α
INF-γ2: Interferon γ2
IRF3: Interferon regulatory factor 3
JNK: C-Jun N-terminal protein kinase
keap1: Kelch-like ECH-associated protein 1
LDH: Lactate dehydrogenase
LEAP: Liver expressed antimicrobial peptide
Lfabp: Liver fatty acid binding protein
LOD: Limit of detection
LOQ: Limit of quantification
LZ: Lysozyme
MAPKs: Mitogen-activated protein kinases
MCH: Mean corpuscular hemoglobin
MCHC: Mean corpuscular hemoglobin concentration
MCV: Mean corpuscular volume
MDA: Malondialdehyde
MLCK: Myosin light chain kinase
ND: Non-detected
NIV-3Glc: Nivalenol-3-glucoside
NM: Non-mentioned
NRE: Nitrogen retention efficiency
Nrf2: Nuclear factor erythroid 2-related factor 2
NTB: Nitroblue tetrazolium assay
OTA: Ochratoxin A
PAPs: Processed animal proteins
PC: Protein carbonyl
PL: Phospholipids
ppar-γ: Peroxisome proliferator-activated receptor
RBCs: Red blood cells
RE: Recovered energy
RF: Relative brood amount
RN: Retained nitrogen
ROS: Reactive oxygen species
SF: Spawning frequency
SGR: Specific growth rate
SOD: Superoxide dismutase
srebp1: Sterol responsive element binding protein 1
SULT: Sulfotransferase
T-2: Toxin T-2
T-2-3Glc: T-2-toxin-3-glucoside
TAOC: Total antioxidant capacity
TGC: Thermal unit growth coefficient
TGs: Triglycerides
TJs: Tight junctions
TNF-α: Tumour necrosis factor α
TP: Total protein
TW: Total weight
UGT: Glucuronosyltransferase
Vtg: Vitellogenin
WBC: White blood cells
WG: Weight gain
ZEN: Zearalenone
ZEN-4Glc: Zearalenone 4-glucoside
α-ZEL: α-Zearalenol
α-ZEL-14Glc: α-Zearalenol-14-glucoside
β-ZEL: β-Zearalenol
β-ZEL-14Glc: β-Zearalenol-14-glucoside
